# Culture Enriched Molecular Profiling of the Cystic Fibrosis Airway Microbiome

**DOI:** 10.1371/journal.pone.0022702

**Published:** 2011-07-28

**Authors:** Christopher D. Sibley, Margot E. Grinwis, Tyler R. Field, Christina S. Eshaghurshan, Monica M. Faria, Scot E. Dowd, Michael D. Parkins, Harvey R. Rabin, Michael G. Surette

**Affiliations:** 1 Department of Microbiology and Infectious Diseases, University of Calgary, Calgary, Alberta, Canada; 2 Department of Medicine, University of Calgary, Calgary, Alberta, Canada; 3 Adult Cystic Fibrosis Clinic, University of Calgary, Calgary, Alberta, Canada; 4 Medical Biofilm Research Institute, Lubbock, Texas, United States of America; 5 Research and Testing Laboratory of the South Plains, Lubbock, Texas, United States of America; 6 Farncombe Family Digestive Health Research Institute, Faculty of Health Sciences, McMaster University, Hamilton, Ontario, Canada; Columbia University, United States of America

## Abstract

The microbiome of the respiratory tract, including the nasopharyngeal and oropharyngeal microbiota, is a dynamic community of microorganisms that is highly diverse. The cystic fibrosis (CF) airway microbiome refers to the polymicrobial communities present in the lower airways of CF patients. It is comprised of chronic opportunistic pathogens (such as *Pseudomonas aeruginosa*) and a variety of organisms derived mostly from the normal microbiota of the upper respiratory tract. The complexity of these communities has been inferred primarily from culture independent molecular profiling. As with most microbial communities it is generally assumed that most of the organisms present are not readily cultured. Our culture collection generated using more extensive cultivation approaches, reveals a more complex microbial community than that obtained by conventional CF culture methods. To directly evaluate the cultivability of the airway microbiome, we examined six samples in depth using culture-enriched molecular profiling which combines culture-based methods with the molecular profiling methods of terminal restriction fragment length polymorphisms and 16S rRNA gene sequencing. We demonstrate that combining culture-dependent and culture-independent approaches enhances the sensitivity of either approach alone. Our techniques were able to cultivate 43 of the 48 families detected by deep sequencing; the five families recovered solely by culture-independent approaches were all present at very low abundance (<0.002% total reads). 46% of the molecular signatures detected by culture from the six patients were only identified in an anaerobic environment, suggesting that a large proportion of the cultured airway community is composed of obligate anaerobes. Most significantly, using 20 growth conditions per specimen, half of which included anaerobic cultivation and extended incubation times we demonstrate that the majority of bacteria present can be cultured.

## Introduction

Defective ion transport across epithelial surfaces results in impaired mucociliary clearance manifesting as an inability to effectively remove bacteria from cystic fibrosis (CF) airways [1] thereby promoting bacterial colonization of a normally sterile site [2]. Traditional culture-dependent approaches have implicated a small number of bacterial pathogens with relevance to CF airway disease [3]; however, the list continues to grow [4]. The classic CF pathogens are representatives of two divisions of bacteria, the Proteobacteria (*Pseudomonas aeruginosa*, *Burkholderia cepacia* complex, *Haemophilus influenzae*, *Stenotrophomonas maltophilia* and *Achromobacter xylosoxidans*) and Firmicutes (*Staphylococcus aureus*) [5]. In recent years, culture-based diagnostics have been augmented with molecular approaches for community profiling [6]–[13] revealing that CF airways, similar to other body sites, harbor numerous organisms that evade detection by routine cultivation [6], [7], [14]–[16]. Although culture-based studies have highlighted the value of using non-conventional approaches [17], there has been considerable interest in adopting culture-independent approaches to better define the ecology of chronic CF airway infections [5], [18].

Culture-independent approaches were pioneered more than 30 years ago [19], [20]. They have since been considered a more objective means to study natural microbial communities because of the apparent limitations of conventional culture techniques [21]–[24]. The explosion in metagenomics data has revolutionized microbial ecology and generated estimates that only 0.001 to 1% of prokaryotes in the environment have been cultured in the laboratory [22], [25]–[27]. This “great plate count anomaly” [27] does not appear to be as dramatic for populations of microbes that inhabit the human body [28]–[31]. Nonetheless the prevailing view is that the majority of bacteria associated with the human microbiome are not readily cultivable.

We have used the complex polymicrobial microbiota associated with CF airways as a means to comprehensively evaluate the utility of using culture-dependent approaches for studying a clinically relevant microbial community [10]. Our data from several years of using more extensive, yet straightforward, cultivation approaches demonstrates that the cultured airway microbiome is far more complex than the view obtained by conventional CF culture methods. To more directly examine the cultivability of the airway microbiome we examined six samples in depth using molecular and culture-based profiling. We demonstrate that by combining culture-dependent and culture-independent approaches (culture-enriched molecular profiling) to study airway microbiology can enhance the sensitivity of either approach alone and that the majority of bacteria present can be cultured.

## Results

### Standard Culture-Dependent Profiling of CF Airway Microbiology

**Figure 1:**
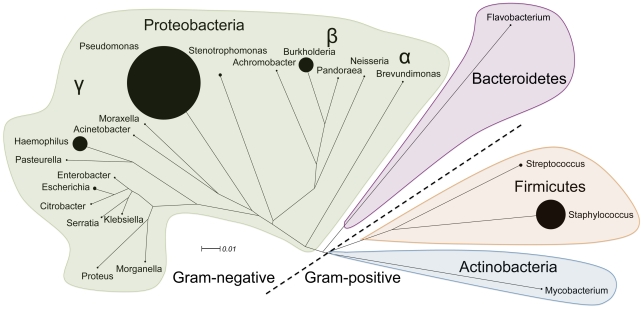
Bacterial genera recovered from CF sputum during 28 years of using conventional cultivation approaches. The proportional abundance of each genus in the culture collection (19 250 isolates) is depicted with solid circles.

Standard CF microbiology culture-based protocols vary slightly between clinical microbiology laboratories but always include a limited number of selective media and growth conditions, and identification is usually limited to specific organisms that are recovered on these media [32]. This strategy effectively recovers the classically recognized principal CF pathogens. The routine cultivation practice in the Southern Alberta Cystic Fibrosis Clinic includes the following agars: CBA, CHOC, MAC, MSA and OFPBL. Throughout the last 28 years in this clinic 19,250 bacterial isolates have been recovered and identified with these culture conditions (an additional 1,891 isolates were identified as fungal species). Members of four phyla have been identified with the majority of isolates belonging to the gamma subdivision of the Proteobacteria (Figure 1). Figure 1 represents the classic view of CF microbiology, whereby organisms belonging to the *Pseudomonas*, *Staphylococcus*, *Burkholderia* and *Haemophilus* genera dominate the microbiology landscape, while the presence of numerous other Gram-negatives, *Streptococcus* and *Mycobacterium* spp. are less common [4].

### Expanded Culture-Dependent Profiling of CF Airway Microbiology

**Figure 2:**
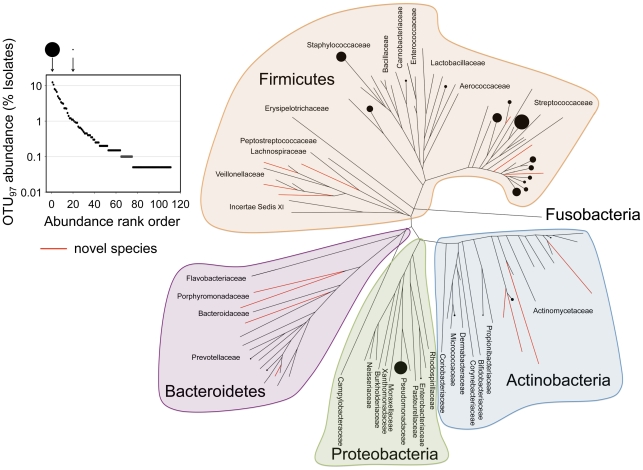
The abundance of 3% OTUs (percent of the total isolates in the culture collection) generated by using non-conventional approaches for microbial cultivation from CF sputum and the phylogenetic relationship between recovered isolates. OTUs that are represented in greater than 1% of the entire culture collection (2 015 isolates) are highlighted on the phylogenetic tree with proportionally sized solid circles according to the legend above the abundance plot. OTUs with less than 97% identity to any 16S rRNA sequence in public databases are indicated with red branches.

Although characterizing the diversity of cultured isolates with a strategy involving recovery of pure cultures may be fundamentally flawed [22], [33], such strategies have made enormous contributions towards understanding the human microbiome [34]–[36]. We used a “colony picking” and purification approach to characterize the organisms that were cultivable but may have been missed or overlooked by standard practices for CF microbiology. Using this approach we purified only colonies that appeared to be morphologically distinct. In addition to the recommended media for CF, we routinely included seven additional solid media and anaerobic incubation was done for many of the cultures (Table S1). During a four-year period (January 2006–January 2010) we examined 351 CF sputum specimens from 117 adult patients attending the Southern Alberta Cystic Fibrosis Clinic (Table S2). We recovered and identified 2,015 isolates by using partial sequence of the 16S rRNA gene. The culture collection could be organized into 110 operational taxonomic units (OTUs; distinct 16S rRNA sequences at a certain-cutoff of sequence diversity [37]) at a clustering threshold of 97% [38] (Table S3). The OTUs (average length 745 bp) could be classified into 33 distinct families from five phlya (Figure 2). There was a significantly different distribution as compared to the conventional perspective (Figure 1), with members of the Firmicutes (low-G+C Gram-positives) being the most common. Thirty-one OTUs were represented by greater than or equal to ten isolates (>0.5% of the culture collection). By far, the most common and diverse family to be cultured was the Streptococcaceae, which included 23 OTUs (993 isolates; 49.3% of the collection). It is clear that the standard species definitions by OTUs may underestimate the number of species in a community [39], [40], particularly in members of the Streptococcaceae [41], [42] where many well-defined species have less than a 3% difference in 16S rRNA gene sequence. Significantly more richness is noted within this family if the clustering threshold is adjusted to 99% (Figure S1).

As expected, the isolation of recognized pathogens such as *P. aeruginosa* and *S. aureus* was very common (10.4 and 7.9% of total isolates, respectively). However, the recovery of organisms that are not reported by using the routine clinical protocols such as members of the Actinomycetaceae, Carnobacteriaceae, Coriobacteriaceae, Enterobacteriaceae, Lactobacillaceae, Micrococcaceae, Prevotellaceae, Propionibacteriaceae and Veillonellaceae were also common (greater than ten isolates each). The culture collection also included 14 OTUs with less than 97% identity to any 16S rRNA sequence in public databases, suggesting that novel phylotypes are present in CF airways.

Comparison of Figure 1 and Figure 2 provides very different perspectives of the CF airway microbiome. It should be noted that the data in these figures represents the occurrence of the different bacteria in the samples analyzed. For each cultured strain in Figure 2 we also have quantitative microbiology and observed that this diversity is also present at high abundance (data not shown). This is consistent with previous studies that have shown that many of these organisms are present at concentrations comparable to the conventional CF pathogens detected by standard clinical microbiology [6], [17].

### Culture-enriched Molecular Profiling of the CF Airway Microbiome

The data used to generate Figure 2 does not represent a concerted effort to exhaustively culture each sample and variable approaches were taken at different times. As such it represents a general survey of cultivable species over a four-year period. Included in this collection are many species previously identified only by culture-independent studies as well as many novel isolates. In order to more accurately assess the cultivable vs non-cultivable organisms in the CF airway microbiome, we examined six clinical samples in depth using direct molecular profiling and culture-enriched molecular profiling.

Using colony morphology as a predictor of genetic relatedness can generate conservative estimates of the accurate cultivable diversity [43]. Furthermore, organisms recovered by mixed enrichment culture are often not recovered by axenic culture [33], [44]. To avoid the pitfalls associated with sampling individual colonies, we chose to utilize Terminal Restriction Fragment Length Polymorphism (T-RFLP) analysis to generate community profiles of complete bacterial populations recovered by culture to directly assess the cultivable vs non-cultivatable bacteria in these samples. T-RFLP is a popular culture-independent technique with the capacity to resolve community members based on the position of restriction sites in the 16S rRNA gene [45] and has been used extensively to profile CF airway communities [5], [15].

Careful attention was made to the design of our culture-enrichment protocol. The time interval between collection of a clinical specimen and entry into an environment of strict anaerobiosis can have a significant impact on recovery efficiencies due to exquisite oxygen sensitivity of some anaerobes [29], [46]. For this reason, fresh sputum was transferred to an oxygen-free atmosphere within two minutes. We used 150 mm Petri plates to increase the available surface area for microbial growth. Culture-dependent approaches are limited because growth on complex media often enriches for community members with an ‘r’-strategy or fast growers (such as *P. aeruginosa*) that overgrow and obscure detection of the slower growing population [10], [47]. To reduce this problem, traditional cultivation techniques can be improved by the addition of inhibitors of certain organisms or antibiotics [48], [49]. The six patients that provided samples for this study are all known to be chronically colonized by *P. aeruginosa*; therefore, many of the media types included colistin sulfate to abrogate overgrowth of this principal pathogen.

**Figure 3:**
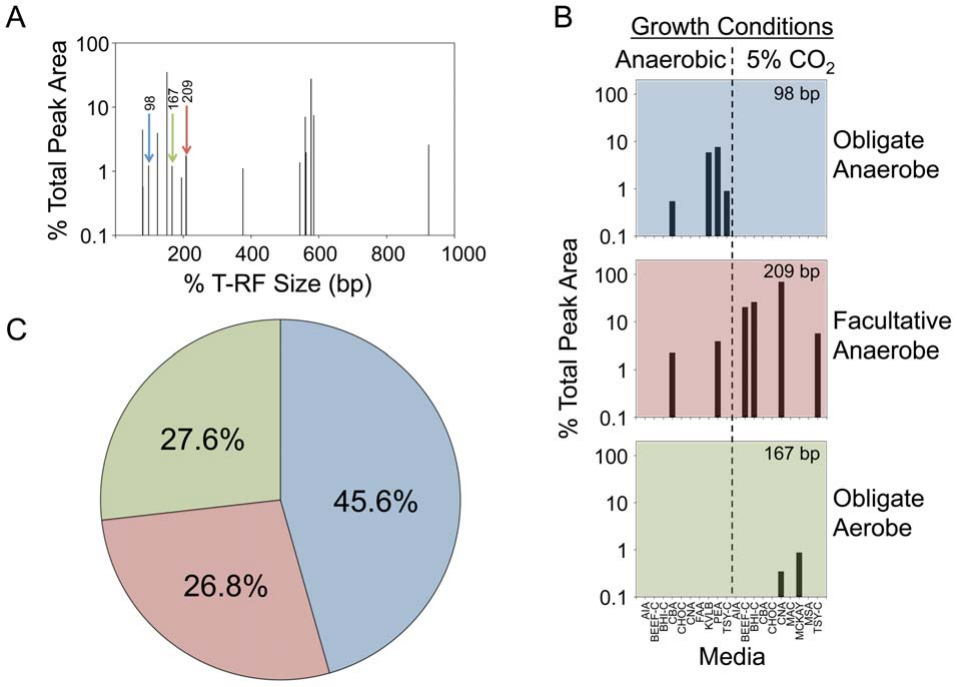
The aerotolerance of the airway microbiome was evaluated by assigning T-RFs to three broad categories representing obligate anaerobes, facultative anaerobes and obligate aerobes. An example of a T-RFLP profile generated directly from sputum is shown (A). The three T-RFs highlighted (size in bp is shown) in the sputum profile provide representative examples of each category, which were determined based on the culture-enrichment conditions under which each T-RF was recovered (B). The overall proportion of each organism category in the six patients investigated is illustrated (C).

We used 20 growth conditions per specimen, half of which included anaerobic cultivation. Routine CF microbiology protocols require cultures to be evaluated at 48 hours [32]; however, we incubated cultures for seven days because increasing incubation times can have a profound effect on recovery efficiencies [50]. Bypassing the requirement for colony purification, we generated community profiles from harvested microbial growth from each plate (examples shown in Figure S2). We determined the aerotolerance of the cultured microbiome by analyzing the incidence of the T-RFs under the conditions tested (Figure 3). Nearly half (114 instances; 45.6%) of the molecular signatures detected by culture from the six patients were only identified in an anaerobic environment, suggesting that a large proportion of the cultured airway community is composed of obligate anaerobes. An additional 67 instances (26.8%) were detected under both anaerobic and microaerophilic conditions, suggesting that the corresponding organisms are facultative anaerobes. Obligate aerobes (only recovered in the presence of 5% CO_2_) made up the remainder of the T-RFs (27.6%; 69 instances).

**Figure 4:**
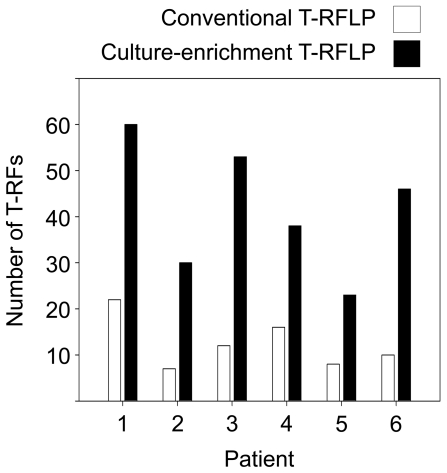
Culture-enrichment increases the number of discernable community members as compared to direct molecular detection from sputum (conventional) by using T-RFLP.

The average number of T-RFs ascertained via direct molecular detection from sputum was 12.5 (+/−2.3). Remarkably, the cultivation-enrichment strategy increased the total number of detectable unique T-RFs by greater than threefold, with an average of 41.6 (+/−5.7) discerned per complete culture set (Figure 4). Equally noteworthy was the observation that of the organisms perceived directly from sputum with the culture-independent approach, 84% (63 of 75 instances) were recovered by at least one culture-enrichment condition. 65.1% of the T-RFs recovered by culture-enrichment could be accounted for by using the conditions recommended for CF (Figure S3); however, most of these organisms do not meet the requirements for identification in the clinical laboratory. Most of the cultivable T-RFs (57 of 63 instances; 90.5%) present in the community profile generated directly from sputum were recovered by using anaerobic conditions. The same could be said of the T-RFs recovered under conditions currently recommended for CF, whereby the majority (37 of 41 instances; 87.8%) was detectable under at least one anaerobic condition. T-RFs corresponding to facultative anaerobes made up the largest class of molecular signatures recovered under standard conditions (26 of 41 instances; 63.4%). Chocolate agar is currently the only anaerobic culture condition recommended for CF microbiology (for purposes of *P. aeruginosa* suppression, not recovery of strict anaerobes [3], [32]), which recovered 45.5% (10 of 22 instances) of all the obligate anaerobes. Conventional cultures also failed to account for the all the T-RFs corresponding to facultative anaerobes or obligate aerobes.

**Figure 5:**
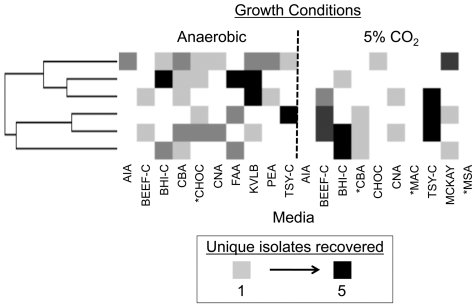
Each patient's sputum required a unique set of culture conditions to represent the complete collection of T-RFs recovered by enrichment. The culture conditions required for inclusive representation of cultivated richness from each sample (shown horizontally) are shaded according to the number of unique T-RFs they recovered according to the legend provided. Culture conditions that are not shaded did not recover unique T-RFs. Culture conditions currently recommended for CF microbiology are indicated with an asterisk.

The culture-enrichment T-RFLP data was used to determine the conditions required for recovery of the complete set of unique T-RFs from each patient (Figure 5). Markedly, the culture conditions were very patient-specific. Between six to ten different conditions per patient were necessary to fully recover community richness. Three culture conditions (two of which are recommended for CF) were redundant and not required by any patient for complete T-RF recovery.

The results of the culture-enrichment T-RFLP suggested that the culture collection we generated by using a colony picking approach (Figure 2) did not comprehensively represent the true richness of the cultivable airway microbiome. Culture-enrichment recovered an average of 5.89 (+/−0.6) T-RFs per culture condition in contrast to an average of 2.2 (+/−0.2) pure isolates recovered using standard microbiological methods (Table S1).

The culture-enriched molecular profiling using T-RFLP analysis also provides a rapid method to find optimal growth conditions for the isolation of specific organisms represented by individual T-RFs. For example, in Figure 3, the facultative anaerobe represented by the T-RF at 209 bp represents about 1% of the bacteria present in sputum (Figure 3A) but represents almost 80% of the bacteria present on the CNA plates incubated in 5% CO_2_ (note that it is not present on CNA plates incubated anaerobically). From the pyrosequencing data (see below) we can identify this as an *Actinomyces* species.

### Culture-enriched Molecular Profiling of the CF Airway Microbiome with Pyrosequencing

Identification of specific T-RFs requires *in silico* prediction, which can be unreliable and ambiguous [51]–[53]. Therefore, we sought to confirm our community profiling data and obtain organism classifications with pyrosequencing. Four patients were further analyzed based on the number of T-RFs identified by direct molecular detection from sputum; two with the greatest and two with the lowest richness (dominated by *P. aeruingosa*) were selected (Figure 4). To improve the dynamic range over that of typical clone libraries, we used Roche's 454 massively parallel sequencing [54] to generate an average of 2,695 filtered sequence reads for each of the enrichment pools that were determined to be essential for inclusive culture-enrichment profiling (Figure 5). Comparison to the corresponding deep sequencing directly from sputum (average 172,355 filtered sequences per sputum) was used to assess cultivability.

The BLAST-based approach to identify genera was validated. Table S4 provides the comparative analysis of pyrosequencing-like ribosomal fragments. It can be seen that the true percentages derived directly from the definition lines of the downloaded dataset and the results of the BLAST analysis show the highest correlation (R^2^) (Table S5). Qiime-based taxonomic classification provided the next highest correlation followed by RDP classification. Correlations of BLAST and Qiime were significant (Table S6). Thus, while classification schemes are of high utility and represent a lower computational burden, making them attractive for large datasets, low-end computation environments and online resources, this validation study demonstrates that BLASTn also provides accurate taxonomic characterization of such data.

**Figure 6:**
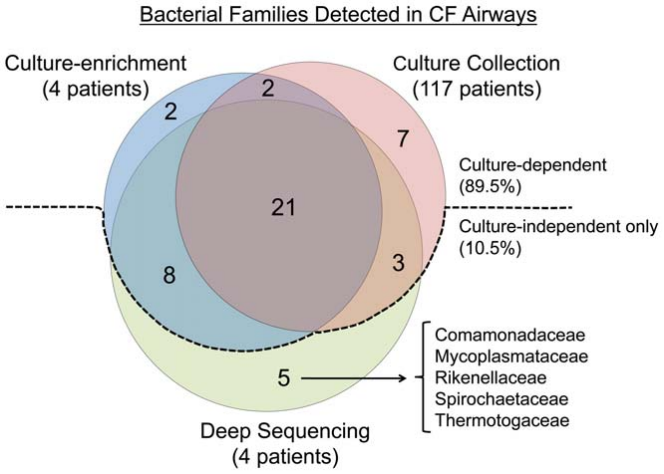
The vast majority of bacterial families detected in this study were detected with a culture-dependent approach. Culture-dependent techniques included culture-enrichment (from four patients) and using expanded culture conditions and picking representative colonies (culture collection from 117 patients). Five families were detected only with a culture-independent approach (deep 16S rRNA sequencing). The families in each sector of the Venn diagram are listed in Table S7.

After removing singletons and doubletons to improve the robustness for comparison between samples [55], 37 bacterial families were detected with the culture-independent deep sequencing from four sputum samples. We used the families recovered in our culture collection (Figure 2) and the families detected from our culture-enrichment conditions as a basis for comparison (Table S1). Twenty-one bacterial families were present in all three datasets (Figure 6). Remarkably, 43 of the 48 families (89.5%) could be accounted for by a culture-dependent means. The families detected with a culture-dependent approach (present in the culture collection and/or were recovered by using enrichment culture) were all members of five phyla (Firmicutes, Bacteroidetes, Proteobacteria, Actinobacteria and Fusobacteria). Though the same phyla were observed between the culture collection and culture-enrichment, ten additional families were recovered by enrichment that had not been recovered throughout our extensive colony-based surveillance including Bacillales Incertae Sedis, Clostridiaceae, Clostridiales Family XIII. Incertae Sedis, Eubacteriaceae, Mycobacteriaceae, Nocardiaceae, Planococcaceae, Rhizobiaceae, Bacteriovoracaceae and Ruminococcaceae. It was enrichment and not deep sequencing that detected Burkholderiaceae and Enterococcaceae.

Five families were only recovered by using the culture-independent approach including Comamonadaceae, Rikenellaceae, Mycoplasmataceae, Spirochaetaceae and Thermotogaceae. It is noteworthy that these five families are from different phyla (Proteobacteria, Bacteroidetes, Tenericutes, Spirochaetes, and Thermotogae, respectively); the latter three phyla were only detected by using a culture-independent approach. All five families were characterized by a low abundance of sequences, each detected in less than 0.002% of total reads.

Human-associated oral microbial communities can be very similar when classified at the level of genus [56]. In CF sputum, an average of 13.5 (+/−1.3) genera were detected per patient sample in greater than 0.001% of the total reads (with singletons and doubletons removed). Thirty-one genera were represented in the complete data set at >0.001% total reads (Figure S4). As predicted by the uniqueness of conditions required to recover maximum richness by enrichment (Figure 5), each patient demonstrated a unique collection of genera. There was significant variability in the relative concentrations of the genera shared between patients, in some cases differing by more than four orders of magnitude. Six genera including *Pseudomonas*, *Prevotella*, *Streptococcus*, *Veillonella*, *Actinomyces* and *Paludibacter* were shared between all patients; however the latter was represented as either a singleton or doubleton in three of the four patients.

**Figure 7:**
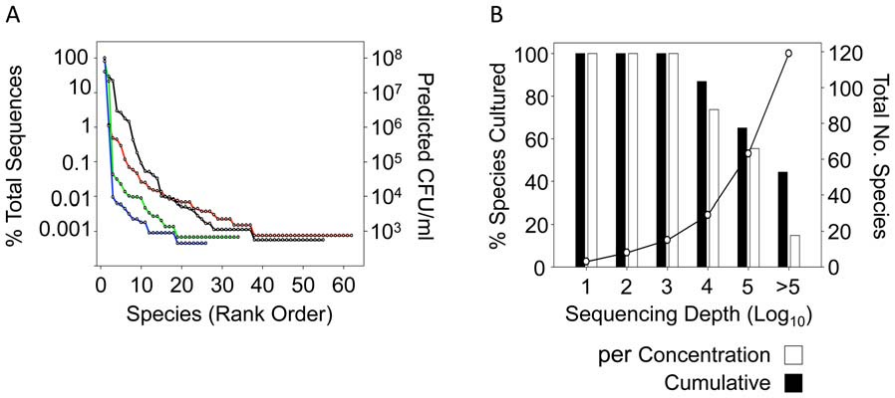
The abundance of each species detected by 16S rRNA sequencing is shown as a rank order by percentage of the total sequences recovered from each patient (A). 16S rRNA sequencing from the enrichment pools was used to determine the percentage of organisms detected by direct molecular detection from sputum that were recovered by culture-enrichment (B). The percentage of cultured species from the four patients is shown as cumulative total as sequencing depth is increased (solid bars) and as a percentage of only the organisms represented at a specific concentration range (open bars).

At the species level, a mean of 44.8 (+/−8.2) different organisms could be discerned per patient with direct 16S rRNA sequencing from sputum. However, due to the characteristic long-tailed distribution (greater diversity at low concentrations) of animal-associated bacterial communities [55], [56], the majority of species (53.9%) were represented as singletons or doubletons (Figure 7A). To what extent these represent true rare species is not clear. It is important to note that pyrosequencing may overestimate the species diversity. An underappreciated problem is that the sequencing error rates are significant. For many applications, such as genome sequencing, the high coverage rate will correct for most sequencing errors. However, in microbial diversity profiling each read represents a single isolate, and as a result the high error rates can lead to overestimation of diversity (see Figure S5). Consequently, these methods are particularly good at profiling distributions of major groups but less effective for accurate species profiling.

With these caveats, we observed that culture-enrichment recovered an average of 8.3 different species per culture condition tested. This confirmed that our culture collection generated by using representative colonies does not reflect the true diversity of cultured organisms, which is consistent with our T-RFLP data. Selecting representative colonies underrepresented diversity by approximately four-fold (a mean of 8.3 different species detected from enrichment pools by 16S rRNA sequencing versus 2.1 different isolates collected per culture condition).

### Cultivated vs Uncultivated Bacteria in the CF Airway Microbiome

One goal of this study was to determine the relative proportions of cultivable bacteria in the CF airways using straightforward methods. Every dominant community member in the four patients (the 13 organisms that could be detected at a depth of 1000 16S rRNA sequences) was recovered by enrichment (Figure 7B). Twenty-seven community members were detectable at a sampling depth of 10^4^ and only four (14.8%) of these species were undetected by culture-enrichment including *Leptotrichia* sp., *Paludibacter* sp., *Tepidimicrobium* sp. and *Neisseria* sp., all of which are members of families with cultured representatives. Further increasing the sampling depth to encompass the organisms represented by 10^5^ 16S rRNA sequences (doubletons in three of the four patients are included at this depth) revealed that 65.1% of the 63 species detected from the four patients were recovered by enrichment. Interestingly, a mean of 4.8 species were detected from each sputum sample only by culture-enrichment.

Conventional T-RFLP is a very useful community profiling technique that has sensitivity for organism prediction equivalent to generating approximately 10^4^ 16S rRNA sequences (Figure S6). By complementing the culture-independent approach with culture-enrichment the sensitivity of the both approaches is significantly improved. The number of distinct organisms detected at the species-level by culture-enrichment was 1.9 (+/−0.3) fold greater than the community size predicted by the number of T-RFs generated from direct detection from sputum (black vs. hatched bars in Figure S6).

## Discussion

The organisms recovered by cultivation in this study were consistent with previous studies, such as the genera reported by molecular and culture-dependent studies and overall aerotolerance [4], [5], [57]. For example, by culture we recovered 14 of the 15 genera and all eight species recently reported to be associated with all the healthy oral cavities examined [56]. We also observed that CF sputum microbiology is very patient-specific and a significant amount of inter-individual variability exists within shared genera. Similar observations have been made for human gut communities [58].

It is no surprise that the reported microbes identified by standard protocols for CF microbiology do not accurately reflect cultivable diversity because the conditions have been optimized to be selective for classic CF pathogens. Although not all conditions are pathogen-selective per se, the clinical laboratory only “works up” recognized CF pathogens. Consistent with previous observations [14], we confirmed that these limited culture conditions have the capacity to recover a fraction (65.1%) of the organisms detected in sputum by using T-RFLP. However, with the addition of straightforward culture conditions to the standard protocol it can increase this proportion to 84%; it is possible that cultivability of the airway microbiome could further be enhanced with supplementary conditions. Organisms detectable with 10^3^ and 10^4^ 16S rRNA sequences were recovered by culture in 100% and 86.8% of instances, respectively. However, the cultivability appears to drop to 65% at a sequencing depth of 10^5^. It is important to consider that the interpretation is complicated by stochastic limits of detection and that the apparent diversity at this level may be exaggerated by errors generated in pyrosequencing. To accurately assess the cultivability for community members at this level, even deeper sequencing would be required to get an accurate representation of community composition. What constitutes the rare microbiome is not easily predicted from deep sequencing alone.

Significant advances in microbial cultivation strategies have dramatically improved our understanding of cultivability [26], [43], [59], [60]; however, to assume that complex and exotic media formulations are required to improve recovery efficiencies is likely not justified [61]. We have shown that the majority of organisms from CF airways can be grown on commercially available media. It is often a daunting task to develop new media [61]. However, if particular organisms are sought, the enrichment data can be used as a starting point to develop more selective conditions for isolation [62]. Culture-enrichment could also be an extremely fruitful technique for investigating rare members of the human microbiome. Employing metagenomics for complete genome sequencing of rare community members in complex samples requires enormous sequencing depth [58]. On the other hand, utilizing culture conditions to enrich and reduce the diversity of the background microbial community could offer a practical solution to studying the rare biosphere.

The nature of the rare biosphere is still being debated [63]–[65]. However, what cannot be contested is the existence of a cell that grows into a microcolony on the surface of an agar plate. Data obtained from microbial cultivation efforts will not only serve as a necessary scaffold for future molecular approaches to studying the human microbiome [36], [58], [66] and become essential for functional studies but will also be invaluable benchmarks for evaluating the quality of new technologies designed to explore the limits of the rare biosphere.

## Materials and Methods

### Cultivation of CF sputa

Collection of sputum samples was obtained with the written informed consent of all study patients. This collaborative research has been granted ethical approval by the Conjoint Health Ethics Board of the Faculties of Medicine, Nursing and Kinesiology, University of Calgary, and the Affiliated Teaching Institutions of the Calgary Zone, Alberta Health Services. Routine clinical microbiology protocols were followed on CF sputum samples in a clinical microbiology laboratory as previously described [62]. Solid media included Columbia blood agar (CBA) (Difco) with 5% sheep blood (Med-Ox), CHOC (GC base (Difco), hemoglobin (Gibco), IsoVitaleX enrichment (BBL)), chocolate agar (CHOC), MacConkey agar (MAC), mannitol-salt agar (MSA) and oxidation-fermentation polymyxin bacitracin lactose (OFPBL) agar. Plates were incubated at 35°C in the presence of 5% CO_2_ for two days with the exception of OFPBL cultures, which were incubated at 30°C and chocolate agar cultures which were incubated anaerobically.

Additional culture media included brain heart infusion (BHI) agar (Bacto) (with and without the addition of colistin sulfate and oxolinic acid to 10 mg/L and 5 mg/L, respectively), Columbia-colisitin-nalidixic acid (CNA) agar (BBL) with 5% sheep blood, fastidious anaerobe agar (FAA; Acumedia) with 5% sheep blood, kanamycin (75 mg/L)–vancomycin (7.5 mg/L) laked blood (5%) agar (KVLB; using FAA as the base), MAC (Difco), McKay agar [62], MSA (BBL), phenylethyl alcohol agar (PEA; BBL) with 5% sheep blood and trypticase soy agar (Difco) supplemented with 0.3% yeast extract (TSY; Bacto). Cultivation was performed in the presence of 5% CO_2_ or in the absence of oxygen in an anaerobic work station (Ruskinn Technology Ltd.) with an atmosphere of 85% N_2_, 10% H_2_ and 5% CO_2_ for four days 37°C.

### Culture-enrichment

For the six patients investigated with culture-enrichment, CF sputum was collected following chest physiotherapy. Upon expectoration it was immediately divided in two; half was transferred to an oxygen-free workstation (within two minutes) to ensure that all processing steps could be carried out under strict anaerobiosis at 37°C. Sputum was sheared by repeated passage through a 1 ml Tuberculin Slip Tip syringe (BD) without a needle. For anaerobic culture serial dilutions were made in trypticase soy broth that had been immediately transferred to an anaerobic atmosphere at 37°C following autoclave sterilization and incubated for 48 hours; the broth was supplemented with yeast extract, L-Cysteine hydrochloride hydrate, hemin and Vitamin K to final concentrations of 3 g/L 0.5 g/L, 10 mg/L and 1 mg/L, respectively. For those samples grown in 5% CO_2_, culture serial dilutions were made in TSY without supplements at room temperature under normal atmospheric conditions. Each pre-warmed plate was inoculated with 400 µl of the 10^−3^ dilution, which was distributed evenly on the surface and incubation was carried out for seven days.

Solid media were prepared as recommended by the manufacturer. For anaerobic culture the agars used included BHI, TSY, CBA, CNA, CHOC, FAA, KVLB, PEA, Actinomycetes isolation agar (AIA; Difco) and cooked meat agar (Beef; Fluka). Beef, BHI and TSY were supplemented with colistin sulfate, L-Cysteine hydrochloride hydrate, hemin and Vitamin K to final concentrations of 10 mg/L, 0.5 g/L, 10 mg/L and 1 mg/L, respectively. AIA, Beef, BHI, CBA, CHOC, CNA, TSY, MAC (Difco), McKay agar and MSA were incubated in 5% CO_2_. Media were autoclave sterilized, cooled to 55°C before the addition of supplements and poured into 150 mm Petri dishes. Prior to inoculation plates were pre-incubated for 24 hours in the environment used for cultivation.

### DNA Extraction

DNA was extracted from sputum through mechanical lysis by bead beating (BioSpec) and phenol∶choloroform extraction as previously described [8]. For cells collected as part of the culture-enrichment pools, 4 ml of 0.85% NaCl was added to the surface of the agar plate and cells were collected by means of resuspending the growth with a sterile loop and removed from the plate by pipetting. Samples were centrifuged at 14,000 rpm for 30 seconds and resuspended in 500 µl RLT buffer (Qiagen, Valencia, CA) with β-mercaptoethanol. A sterile 5 mm steel bead (Qiagen, Valencia, CA) and 500 µl of sterile 0.1 mm glass beads (Scientific Industries, Inc., NY, USA) were added for complete bacterial lysis in a Qiagen TissueLyser (Qiagen, Valencia, CA), run at 30 Hz for 5 min. Samples were centrifuged briefly and 100 µl of 100% ethanol was added to a 100 µl aliquot of the sample supernatant. This mixture was added to a DNA spin column, and DNA recovery protocols were followed as instructed in the QIAamp DNA Mini Kit (Qiagen, Valencia, CA) starting at step five of the Tissue Protocol. DNA was eluted from the column with 30 µl of water and samples were diluted accordingly to a final concentration of 20 ng/µl. DNA samples were quantified using a Nanodrop spectrophotometer (Nyxor Biotech, Paris, France).

### Strain identification and phylogenetic analysis

Colony morphology was assessed visually with a magnification lamp; morphologically distinct colonies were streak purified three times under the same conditions from which they were recovered. DNA template was prepared from fresh colonies by re-suspending single colonies in 50 µl of dH_2_O, boiling for 15 minutes and removing cellular debris by centrifugation. Partial 16S rRNA sequence was PCR amplified by using primers 8f (5′-AGAGTTTGATCCTGGCTCAG-3′) [22] and 926r (5′-CCGTCAATTCCTTTRAGTTT-3′) [67]. PCR products were sequenced by Macrogen (Korea) in the forward direction and taxonomic identification was made by using BLAST results against the RDP database (http://rdp.cme.msu.edu/) and the Human Oral Microbiome Database (http://www.homd.org/) and manual assignment. Sequences >400 bp were used and 16S rRNA sequences with >97% identity over the aligned length excluding gaps and non-AGCTU were identified to the species level. Verification that assignments to species did not conflict with the higher level assignments by the RDP classification algorithm was done using a naïve Bayesian rRNA classifier [68]. Operational Taxonomic Units (OTUs) were determined for cultured isolates by using CD-HIT-EST at a clustering threshold of 97% and local alignment of 0.7 and 0.01 for short and longest representative sequences, respectively [69], [70]. Multiple alignments using the longest representative 16S rRNA sequence for each OTU was done using NAST [71]. Phylogenetic trees were constructed using the Neighbor-Joining method and the confidence of the resultant trees assessed using a bootstrap test with 1000 replicates by using the MEGA4 software package [72]. Dendrograms were further manipulated by using SplitsTree4 version 4.10 [73].

### Terminal Restriction Fragment Length Polymorphism Analysis

PCR products were generated as previously described [10] using the 8f primer labeled with VIC (Applied Biosystems). DNA Clean and Concentrator 5 columns (Zymo Research) were used to desalt amplicons before and after restriction digestion with CfoI (Roche). Digestions were carried out for at least 7 hours at 37°C with 200 ng of amplicon and 20 U of restriction enzyme. Capillary electrophoresis was carried out as previously described with the LIZ1200 size standard (Applied Biosystems) [10]. The total fluorescence signal corresponding to the area under all of the peaks for each sample was determined with the GeneMapper software package (Applied Biosystems) by using only terminal restriction fragments (T-RFs) greater than 50 bp in size. Each T-RF was expressed as a percentage of total fluorescence with a threshold detection of 0.1%.

### Massively parallel bTEFAP titanium sequencing

Bacterial tag-encoded FLX amplicon pyrosequencing (bTEFAP) was performed as described previously [74]–[81]. The new bacterial tag-encoded FLX-Titanium amplicon pyrosequencing (bTETAP) approach is based upon similar principles to bTEFAP but utilizes Titanium reagents and Titanium procedures and a one-step PCR, mixture of Hot Start and Hot Start high fidelity Taq polymerases, and amplicons originating from the 27F region numbered in relation to *E. coli* rRNA. The bTEFAP procedures were performed at the Research and Testing Laboratory (Lubbock, TX) based upon RTL protocols (www.researchandtesting.com).

### Bacterial Diversity Analysis

Following sequencing, all failed sequence reads, low quality sequence ends and tags were removed and sequences were depleted of any non-bacterial ribosome sequences and chimeras using custom software described previously [74]–[82] and the Black Box Chimera Check software B2C2 (described and freely available at http://www.researchandtesting.com/B2C2.html). Sequences of less than 350 bp were removed. To determine the identity of bacteria in the remaining sequences, sequences were first queried using a distributed BLASTn.NET algorithm [83] against a database of high quality 16S rRNA bacterial sequences derived from NCBI. Database sequences were characterized as high quality based upon criteria similar to that utilized by RDP ver 9 [84] and included near full length ribosomal sequence that were annotated with valid taxonomic lineages, which did not have degenerate base calls and when aligned with other sequences matched annotated taxonomy designations. Sequences which did not fulfill these criteria were removed to a manual curation process or depleted from the database. Using a .NET and C# analysis pipeline the resulting BLASTn outputs were compiled and validated using sequence identity methods; data reduction analysis was performed as described previously [74]–[82]. The bacteria were classified at the appropriate taxonomic levels based upon the following criteria: sequences with identity scores greater than 97% (<3% divergence) to known or well characterized 16S sequences were resolved at the species level, between 95% and 97% at the genus level, between 90% and 95% at the family level and between 80% and 90% at the order level. After resolving based upon these parameters, the percentage of each bacterial ID was individually analyzed for each sample by providing abundance information based upon relative numbers of reads within a given sample. Evaluations presented at a given taxonomic level, except species level, represent all sequences resolved to their primary genera identification or their closest relative.

### BLAST Validation

A validation of the BLAST-based approach to identify genera was done by using a set of 16S rRNA sequence data characterized as high quality >1300 bp from the RDP database. A query dataset was derived directly from this dataset. Using C# scripts a total of 11,608 sequences were selected randomly. These randomly chosen query sequences were trimmed to 450 corresponding to the region of the *E. coli* ribosome numbered 104F-530R, which corresponds to the average read length of pyrosequencing. The sequence identities were compiled based upon the definition lines derived from the original download to provide relative percentages of each genus within the query set. This was considered the dataset “truth percentages”. As noted this truth data was derived directly from the RDP downloaded dataset. This query set was processed using Qiime [85], RDP classification [86], and BLASTn [83]. The BLAST database was against the Research and Testing Blast database (v 01-01-2011) containing >360 K sequences and processed using Krakenblast (www.krakenblast.com). The genera identifications were then compiled into relative percentages of each genus directly from the Qiime, RDP and BLAST output.

## Supporting Information

Figure S1 The 993 isolates cultured from CF airways that belong to the Streptococcaceae family can be organized into 88 1% OTUs. The proportion of each OTU is depicted on the phylogenetic tree with proportionally sized solid circles according to the provided legend. OTUs with less than 97% identity to any 16S rRNA sequence in public databases are indicated with red branches.
(TIF)

Figure S2 Examples of representative culture enrichment data are provided as pictures of 5% CO_2_ and anaerobic CNA culture plates from one patient (A). The corresponding T-RFLP profiles are shown next to cultures that were collected to generate the enrichment pools (B).
(TIF)

Figure S3 The majority of T-RFs detected directly from CF sputum can be recovered by culture-enrichment. A significant proportion of the T-RFs detected by culture-enrichment were only recovered under non-conventional culture conditions; the proportion of each T-RFs category (obligate anaerobes, facultative anaerobes, obligate aerobes) detected on media recommended for CF microbiology are highlighted in green.
(TIF)

Figure S4 The most abundant genera detected from CF sputum by using deep 16S rRNA sequencing. The total number of sequences corresponding to each genus from all four patients was used to place genera in abundance rank order. Genera detected in greater than 0.001% of total sequences (greater than or equal to seven sequences in the total 689,422 generated from four patients) are shown. For each patient (red, green, yellow and black circles each illustrate an individual) the percent of the total patient-specific sequences represented by each genus is plotted.
(TIF)

Figure S5 Pyrosequencing error results in a broader than expected distribution in 16S rRNA gene sequences and greater apparent sequence diversity. **A.** Distribution of *P. aeruginosa* 16S rRNA gene sequences pooled from the 4 independent 454 pyrosquencing runs of sputum samples. Theoretically all reads should be close to 100% match, however, the deviation from this represents sequencing error. Note this is for samples that have been filtered for poor sequence quality and chimeras. For these samples the estimated error rate is 0.9%. **B.** Cumulative frequency histogram for the *P. aeruginosa* sequences from each of the 4 independent 454 pyrosquencing runs of sputum samples along with the all of the *P. aeruginosa* 16S rRNA genes sequences from isolated organisms obtained by Sanger sequencing (n = 214). Note all four different pyrosequencing samples have similar distributions and only about 27% of the sequences fall in the 99.5–100% percentile (of sequence identity) compared to 85% of the Sanger sequenced samples. If the sequence diversity from the pyrosequencing for *P. aeruginosa* represented the true sequence variability, the cumulative histogram profiles would be expected to be different in each patient sample reflecting individual variability.
(TIF)

Figure S6 A comparison between the numbers of predicted organisms per sputum sample measured by conventional T-RFLP (determined by the number of T-RFs detected), culture-enrichment (as determined by 16S rRNA sequencing from enrichment pools) and by direct 16S rRNA sequencing from sputum at various depths (10^3^ to 10^5^ sequences per sputum).
(TIF)

Table S1 Culture conditions used to generate the isolate collection.
(DOC)

Table S2 Characteristics of the culture collection generated by expanded culturing approaches (January 2006 to January 2010).
(DOC)

Table S3 Operational taxonomic units (3%) identified in the culture collection.
(DOCX)

Table S4 Taxonomic assignment results. Relative percentage of known (truth) dataset using 3 common taxonomic identification methods.
(DOCX)

Table S5 Correlation R2 values of taxonomic assignment based upon a known dataset.
(DOCX)

Table S6 Correlation analysis P-value for taxonomic assignment based upon a known dataset.
(DOCX)

Table S7 Bacterial families detected in this study.
(DOC)

## References

[1] Matsui H, Grubb BR, Tarran R, Randell SH, Gatzy JT (1998). Evidence for periciliary liquid layer depletion, not abnormal ion composition, in the pathogenesis of cystic fibrosis airways disease.. Cell.

[2] Accurso FJ (1997). Early pulmonary disease in cystic fibrosis.. Curr Opin Pulm Med.

[3] Miller MB, Gilligan PH (2003). Laboratory aspects of management of chronic pulmonary infections in patients with cystic fibrosis.. J Clin Microbiol.

[4] Lipuma JJ (2010). The changing microbial epidemiology in cystic fibrosis.. Clin Microbiol Rev.

[5] Rogers GB, Carroll MP, Bruce KD (2009). Studying bacterial infections through culture-independent approaches.. J Med Microbiol.

[6] Harris JK, De Groote MA, Sagel SD, Zemanick ET, Kapsner R (2007). Molecular identification of bacteria in bronchoalveolar lavage fluid from children with cystic fibrosis.. Proc Natl Acad Sci U S A.

[7] Bittar F, Richet H, Dubus JC, Reynaud-Gaubert M, Stremler N (2008). Molecular detection of multiple emerging pathogens in sputa from cystic fibrosis patients.. PLoS ONE.

[8] Rogers GB, Carroll MP, Serisier DJ, Hockey PM, Jones G (2004). Characterization of bacterial community diversity in cystic fibrosis lung infections by use of 16S ribosomal DNA terminal restriction fragment length polymorphism profiling.. J Clin Microbiol.

[9] Rogers GB, Hart CA, Mason JR, Hughes M, Walshaw MJ (2003). Bacterial diversity in cases of lung infection in cystic fibrosis patients: 16S ribosomal DNA (rDNA) length heterogeneity PCR and 16S rDNA terminal restriction fragment length polymorphism profiling.. J Clin Microbiol.

[10] Sibley CD, Parkins MD, Rabin HR, Duan K, Norgaard JC (2008). A polymicrobial perspective of pulmonary infections exposes an enigmatic pathogen in cystic fibrosis patients.. Proc Natl Acad Sci U S A.

[11] Klepac-Ceraj V, Lemon KP, Martin TR, Allgaier M, Kembel SW (2010). Relationship between cystic fibrosis respiratory tract bacterial communities and age, genotype, antibiotics and *Pseudomonas aeruginosa*.. Environ Microbiol.

[12] Kolak M, Karpati F, Monstein HJ, Jonasson J (2003). Molecular typing of the bacterial flora in sputum of cystic fibrosis patients.. Int J Med Microbiol.

[13] Ecker DJ, Sampath R, Massire C, Blyn LB, Hall TA (2008). Ibis T5000: a universal biosensor approach for microbiology.. Nat Rev Microbiol.

[14] Rogers GB, Daniels TW, Tuck A, Carroll MP, Connett GJ (2009). Studying bacteria in respiratory specimens by using conventional and molecular microbiological approaches.. BMC Pulm Med.

[15] Sibley CD, Rabin H, Surette MG (2006). Cystic fibrosis: a polymicrobial infectious disease.. Future Microbiol.

[16] Parkins MD, Sibley CD, Surette MG, Rabin HR (2008). The *Streptococcus milleri* group–an unrecognized cause of disease in cystic fibrosis: a case series and literature review.. Pediatr Pulmonol.

[17] Tunney MM, Field TR, Moriarty TF, Patrick S, Doering G (2008). Detection of anaerobic bacteria in high numbers in sputum from patients with cystic fibrosis.. Am J Respir Crit Care Med.

[18] Raoult D, Fournier PE, Drancourt M (2004). What does the future hold for clinical microbiology?. Nat Rev Microbiol.

[19] Woese CR, Fox GE (1977). Phylogenetic structure of the prokaryotic domain: the primary kingdoms.. Proc Natl Acad Sci of the U S A.

[20] Pace NR, Stahl DA, Lane DJ, Olsen GJ (1986). The analysis of natural microbial populations by ribosomal RNA sequences.. Adv in Microb Ecol.

[21] Ward DM, Weller R, Bateson MM (1990). 16S ribosomal RNA sequences reveal numerous uncultured microorganisms in a natural community.. Nature.

[22] Amann RI, Ludwig W, Schleifer KH (1995). Phylogenetic identification and in situ detection of individual microbial cells without cultivation.. Microbiol Rev.

[23] Rappe MS, Giovannoni SJ (2003). The uncultured microbial majority.. Ann Rev Microbiol.

[24] Keller M, Zengler K (2004). Tapping into microbial diversity.. Nat Rev Microbiol.

[25] Hugenholtz P, Tyson GW (2008). Microbiology - Metagenomics.. Nature.

[26] Alain K, Querellou J (2009). Cultivating the uncultured: limits, advances and future challenges.. Extremophiles.

[27] Staley JT, Konopka A (1985). Measurement of in situ activities of nonphotosynthetic microorganisms in aquatic and terrestrial habitats.. Ann Rev Microbiol.

[28] Socransky SS, Gibbons RJ, Dale AC, Bortnick L, Rosenthal E (1963). The microbiota of the gingival crevice area of man – I: Total microscopic and viable counts and counts of specific organisms.. Arch Oral Biol.

[29] Gordon DF, Stutman M, Loesche WJ (1971). Improved isolation of anaerobic bacteria from gingival crevice area of man.. Appl Microbiol.

[30] Moore WEC, Holdeman LV (1974). Human fecal flora: the normal flora of 20 Japanese-Hawaiians.. Appl Microbiol.

[31] Wilson KH, Blitchington RB (1996). Human colonic biota studied by ribosomal DNA sequence analysis.. Appl Environ Microbiol.

[32] Gilligan PH, Kiska DL, Appleman MD (2006). Cumitech: cystic fibrosis microbiology.

[33] Kopke B, Wilms R, Engelen B, Cypionka H, Sass H (2005). Microbial diversity in coastal subsurface sediments: a cultivation approach using various electron acceptors and substrate gradients.. Appl Environ Microbiol.

[34] Finegold SM, Sutter VL, Mathison GE, Hentges DJ (1983). Normal indigenous flora.. Human intestinal microflora in health and disease.

[35] Moore WE, Moore LV (1994). The bacteria of periodontal diseases.. Periodontol 2000.

[36] Turnbaugh PJ, Ley RE, Hamady M, Fraser-Liggett CM, Knight R (2007). The human microbiome project.. Nature.

[37] Achtman M, Wagner M (2008). Microbial diversity and the genetic nature of microbial species.. Nat Rev Microbiol.

[38] Stackebrandt E, Goebel BM (1994). A place for DNA-DNA reassociation and 16S ribosomal RNA sequence analysis in the present species definition in bacteriology.. Int J Syst Bacteriol.

[39] Tyson GW, Chapman J, Hugenholtz P, Allen EE, Ram RJ (2004). Community structure and metabolism through reconstruction of microbial genomes from the environment.. Nature.

[40] Venter JC, Remington K, Heidelberg JF, Halpern AL, Rusch D (2004). Environmental genome shotgun sequencing of the Sargasso Sea.. Science.

[41] Facklam R (2002). What happened to the streptococci: overview of taxonomic and nomenclature changes.. Clin Microbiol Rev.

[42] Kawamura Y, Hou XG, Sultana F, Miura H, Ezaki T (1995). Determination of 16S rRNA sequences of *Streptococcus mitis* and *Streptococcus gordonii* and phylogenetic relationships among members of the genus *Streptococcus*.. Int J Syst Bacteriol.

[43] Kaeberlein T, Lewis K, Epstein SS (2002). Isolating “uncultivable” microorganisms in pure culture in a simulated natural environment.. Science.

[44] D'Onofrio A, Crawford JM, Stewart EJ, Witt K, Gavrish E (2010). Siderophores from neighboring organisms promote the growth of uncultured bacteria.. Chem Biol.

[45] Liu WT, Marsh TL, Cheng H, Forney LJ (1997). Characterization of microbial diversity by determining terminal restriction fragment length polymorphisms of genes encoding 16S rRNA.. Appl Environ Microbiol.

[46] Flint HJ, Duncan SH, Scott KP, Louis P (2007). Interactions and competition within the microbial community of the human colon: links between diet and health.. Environ Microbiol.

[47] Watve M, Shejval V, Sonawane C, Rahalkar M, Matapurkar A (2000). The ‘K’ selected oligophilic bacteria: A key to uncultured diversity?. Current Science.

[48] Leadbetter JR (2003). Cultivation of recalcitrant microbes: cells are alive, well and revealing their secrets in the 21st century laboratory.. Curr Opin Microbiol.

[49] Konneke M, Bernhard AE, de la Torre JR, Walker CB, Waterbury JB (2005). Isolation of an autotrophic ammonia-oxidizing marine archaeon.. Nature.

[50] Sait M, Hugenholtz P, Janssen PH (2002). Cultivation of globally distributed soil bacteria from phylogenetic lineages previously only detected in cultivation-independent surveys.. Environ Microbiol.

[51] Tu O, Knott T, Marsh M, Bechtol K, Harris D (1998). The influence of fluorescent dye structure on the electrophoretic mobility of end-labeled DNA.. Nucleic Acids Research.

[52] Kaplan CW, Kitts CL (2003). Variation between observed and true Terminal Restriction Fragment length is dependent on true TRF length and purine content.. J Microbiol Methods.

[53] Schutte UME, Abdo Z, Bent SJ, Shyu C, Williams CJ (2008). Advances in the use of terminal restriction fragment length polymorphism (T-RFLP) analysis of 16S rRNA genes to characterize microbial communities.. Appl Microbiol Biotechnol.

[54] Margulies M, Egholm M, Altman WE, Attiya S, Bader JS (2005). Genome sequencing in microfabricated high-density picolitre reactors.. Nature.

[55] Dethlefsen L, Huse S, Sogin ML, Relman DA (2008). The Pervasive Effects of an Antibiotic on the Human Gut Microbiota, as Revealed by Deep 16S rRNA Sequencing.. PLoS Biology.

[56] Bik EM, Long CD, Armitage GC, Loomer P, Emerson J (2010). Bacterial diversity in the oral cavity of 10 healthy individuals.. ISME J.

[57] Manganiello AD, Socransky SS, Smith C, Propas D, Oram V (1977). Attempts to increase viable count recovery of human supragingival dental plaque.. J Periodontal Res.

[58] Qin JJ, Li RQ, Raes J, Arumugam M, Burgdorf KS (2010). A human gut microbial gene catalogue established by metagenomic sequencing.. Nature.

[59] Ingham CJ, Sprenkels A, Bomer J, Molenaar D, van den Berg A (2007). The micro-Petri dish, a million-well growth chip for the culture and high-throughput screening of microorganisms.. Proc Natl Acad Sci U S A.

[60] Zengler K, Toledo G, Rappe M, Elkins J, Mathur EJ (2002). Cultivating the uncultured.. Proc Natl Acad Sci U S A.

[61] Zengler K (2009). Central role of the cell in microbial ecology.. Microbiol Mol Biol Rev.

[62] Sibley CD, Grinwis M, Field TR, Parkins MD, Noorgard JC (2010). McKay Agar Enables Routine Quantification of the *Streptococcus milleri* Group in Cystic Fibrosis Patients.. J Med Microbiol.

[63] Quince C, Lanzen A, Curtis TP, Davenport RJ, Hall N (2009). Accurate determination of microbial diversity from 454 pyrosequencing data.. Nat Methods.

[64] Reeder J, Knight R (2009). The ‘rare biosphere’: a reality check.. Nat Methods.

[65] Kunin V, Engelbrektson A, Ochman H, Hugenholtz P (2010). Wrinkles in the rare biosphere: pyrosequencing errors can lead to artificial inflation of diversity estimates.. Environ Microbiol.

[66] Turnbaugh PJ, Quince C, Faith JJ, McHardy AC, Yatsunenko T (2010). Organismal, genetic, and transcriptional variation in the deeply sequenced gut microbiomes of identical twins.. Proc Natl Acad Sci U S A.

[67] Muyzer G, Teske A, Wirsen CO, Jannasch HW (1995). Phylogenetic relationships of Thiomicrospira species and their identification in deep-sea hydrothermal vent samples by denaturing gradient gel electrophoresis of 16S rDNA fragments.. Arch Microbiol.

[68] Wang Q, Garrity GM, Tiedje JM, Cole JR (2007). Naive Bayesian classifier for rapid assignment of rRNA sequences into the new bacterial taxonomy.. Applied and Environmental Microbiology.

[69] Huang Y, Niu B, Gao Y, Fu L, Li W (2010). CD-HIT Suite: a web server for clustering and comparing biological sequences.. Bioinformatics.

[70] Li W, Godzik A (2006). Cd-hit: a fast program for clustering and comparing large sets of protein or nucleotide sequences.. Bioinformatics.

[71] DeSantis TZ, Hugenholtz P, Keller K, Brodie EL, Larsen N (2006). NAST: a multiple sequence alignment server for comparative analysis of 16S rRNA genes.. Nucleic Acids Res.

[72] Tamura K, Dudley J, Nei M, Kumar S (2007). MEGA4: Molecular Evolutionary Genetics Analysis (MEGA) software version 4.0.. Mol Biol Evol.

[73] Huson DH, Bryant D (2006). Application of phylogenetic networks in evolutionary studies.. Mol Biol Evol.

[74] Callaway TR, Dowd SE, Wolcott RD, Sun Y, McReynolds JL (2009). Evaluation of the bacterial diversity in cecal contents of laying hens fed various molting diets by using bacterial tag-encoded FLX amplicon pyrosequencing.. Poult Sci.

[75] Dowd SE, Callaway TR, Wolcott RD, Sun Y, McKeehan T (2008). Evaluation of the bacterial diversity in the feces of cattle using 16S rDNA bacterial tag-encoded FLX amplicon pyrosequencing (bTEFAP).. BMC Microbiol.

[76] Dowd SE, Sun Y, Secor PR, Rhoads DD, Wolcott BM (2008). Survey of bacterial diversity in chronic wounds using pyrosequencing, DGGE, and full ribosome shotgun sequencing.. BMC Microbiol.

[77] Dowd SE, Sun Y, Wolcott RD, Domingo A, Carroll JA (2008). Bacterial tag-encoded FLX amplicon pyrosequencing (bTEFAP) for microbiome studies: bacterial diversity in the ileum of newly weaned Salmonella-infected pigs.. Foodborne Pathog Dis.

[78] Dowd SE, Wolcott RD, Sun Y, McKeehan T, Smith E (2008). Polymicrobial nature of chronic diabetic foot ulcer biofilm infections determined using bacterial tag encoded FLX amplicon pyrosequencing (bTEFAP).. PLoS One.

[79] Leake JL, Dowd SE, Wolcott RD, Zischkau AM (2009). Identification of yeast in chronic wounds using new pathogen-detection technologies.. J Wound Care.

[80] Suchodolski JS, Dowd SE, Westermarck E, Steiner JM, Wolcott RD (2009). The effect of the macrolide antibiotic tylosin on microbial diversity in the canine small intestine as demonstrated by massive parallel 16S rRNA gene sequencing.. BMC Microbiol.

[81] Wolcott RD, Gontcharova V, Sun Y, Zischakau A, Dowd SE (2009). Bacterial diversity in surgical site infections: not just aerobic cocci any more.. J Wound Care.

[82] Wolcott RD, Gontcharova V, Sun Y, Dowd SE (2009). Evaluation of the bacterial diversity among and within individual venous leg ulcers using bacterial tag-encoded FLX and titanium amplicon pyrosequencing and metagenomic approaches.. BMC Microbiol.

[83] Dowd SE, Zaragoza J, Rodriguez JR, Oliver MJ, Payton PR (2005). Windows .NET Network Distributed Basic Local Alignment Search Toolkit (W.ND-BLAST).. BMC Bioinformatics.

[84] Cole JR, Wang Q, Cardenas E, Fish J, Chai B (2009). The Ribosomal Database Project: improved alignments and new tools for rRNA analysis.. Nucleic Acids Res.

[85] Caporaso JG, Kuczynski J, Stombaugh J, Bittinger K, Bushman FD (2010). QIIME allows analysis of high-throughput community sequencing data.. Nat Methods.

[86] Cole JR, Chai B, Farris RJ, Wang Q, Kulam SA (2005). The Ribosomal Database Project (RDP-II): sequences and tools for high-throughput rRNA analysis.. Nucleic Acids Res.

