# Supplementary figures and images for: Culture Enriched Molecular Profiling of the Cystic Fibrosis Airway Microbiome
Source: PLoS One. 2011 Jul 28;6(7):e22702. doi: 10.1371/journal.pone.0022702 (PMC3145661; doi:10.1371/journal.pone.0022702)

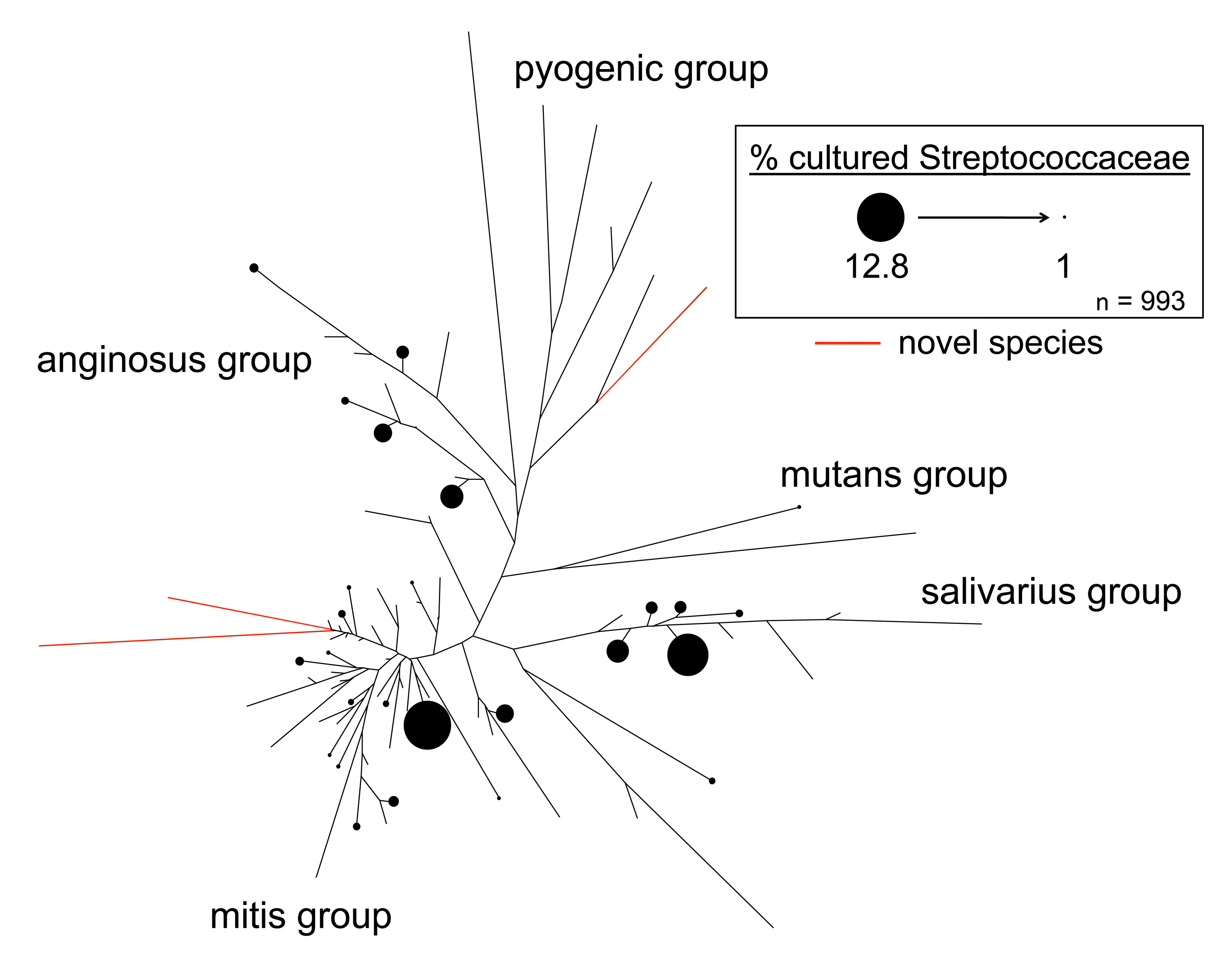

Supplement: Figure S1 — The 993 isolates cultured from CF airways that belong to the Streptococcaceae family can be organized into 88 1% OTUs. The proportion of each OTU is depicted on the phylogenetic tree with proportionally sized solid circles according to the provided legend. OTUs with less than 97% identity to any 16S rRNA sequence in public databases are indicated with red branches. (TIF) [file pone.0022702.s001.tif]

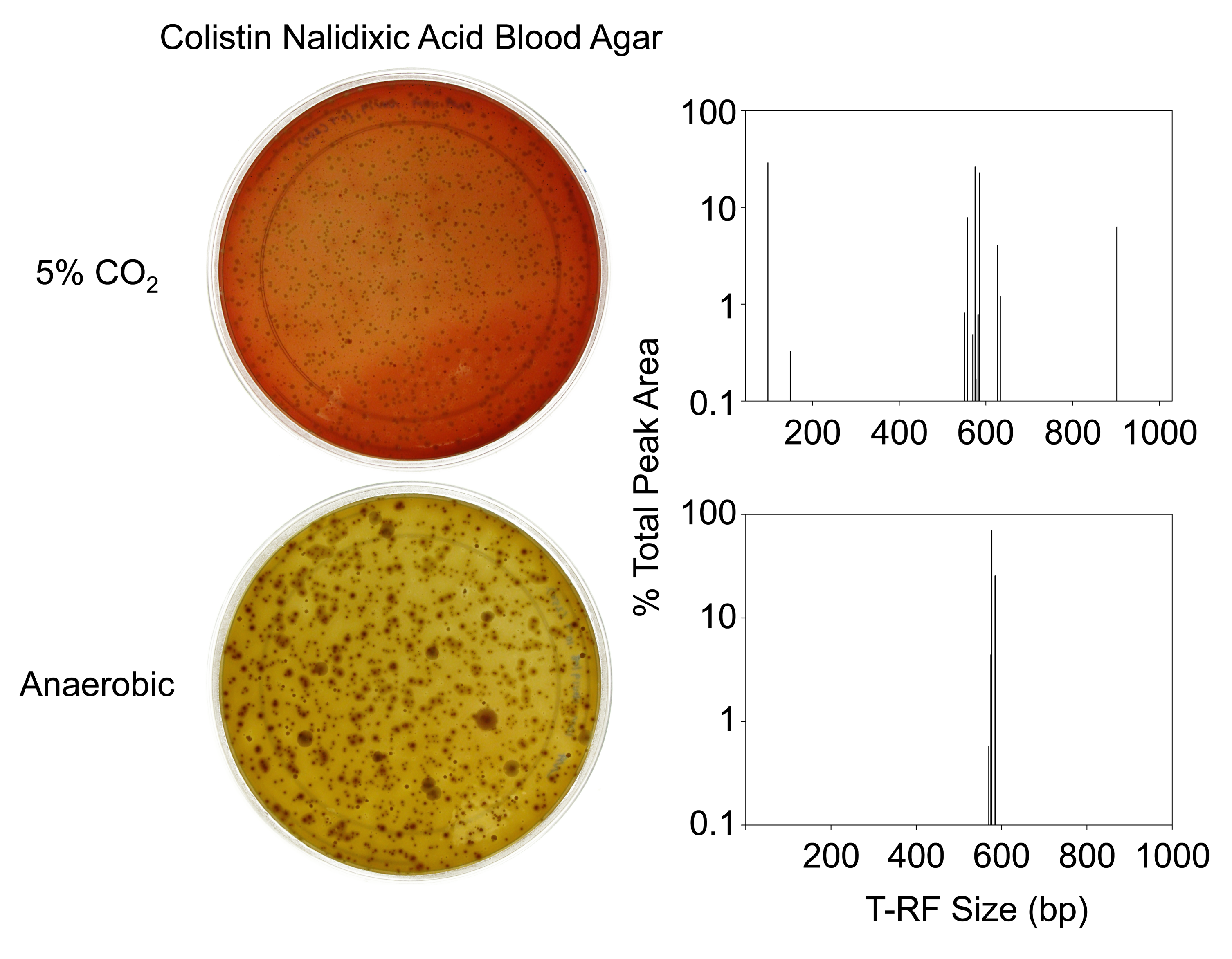

Supplement: Figure S2 — Examples of representative culture enrichment data are provided as pictures of 5% CO2 and anaerobic CNA culture plates from one patient (A). The corresponding T-RFLP profiles are shown next to cultures that were collected to generate the enrichment pools (B). (TIF) [file pone.0022702.s002.tif]

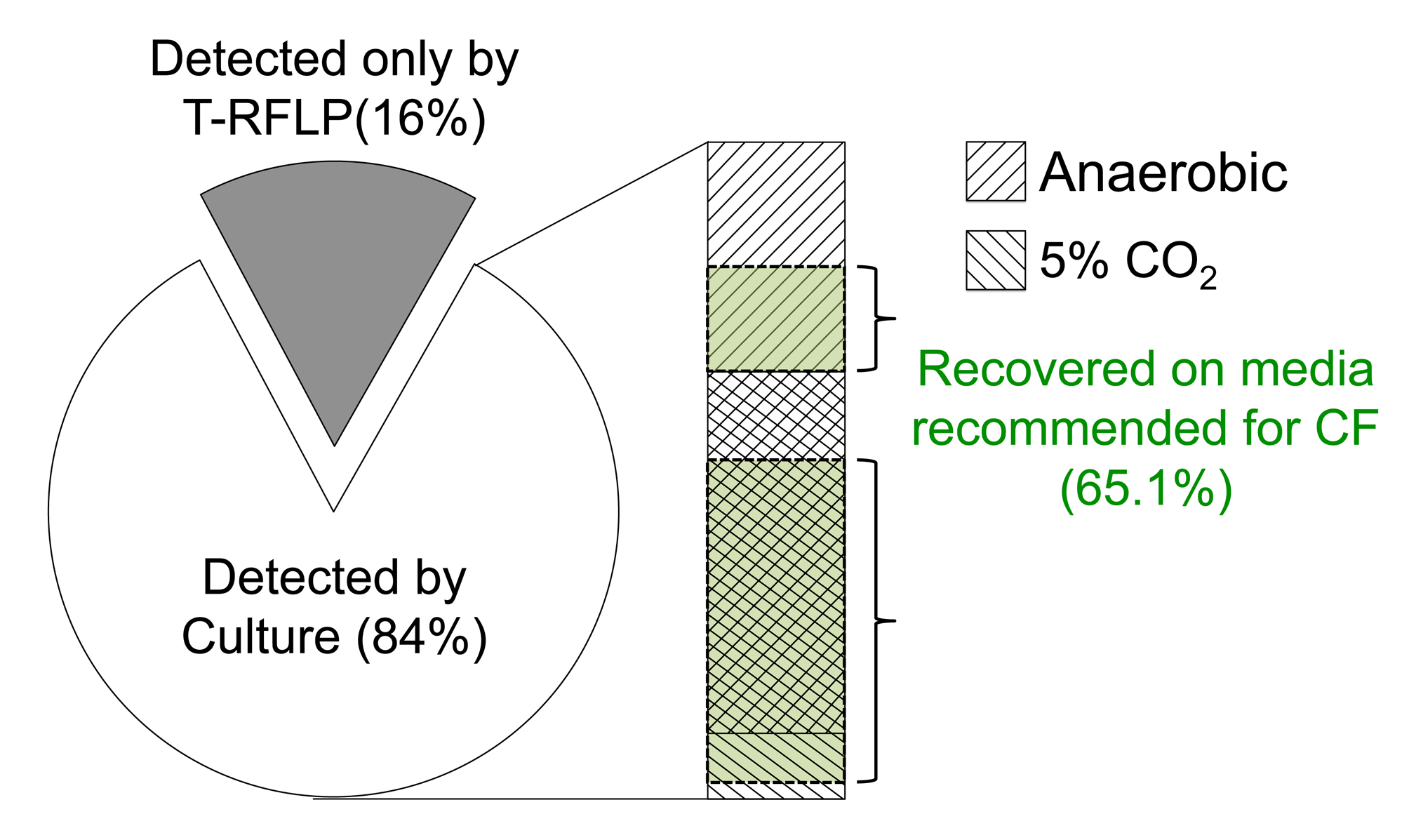

Supplement: Figure S3 — The majority of T-RFs detected directly from CF sputum can be recovered by culture-enrichment. A significant proportion of the T-RFs detected by culture-enrichment were only recovered under non-conventional culture conditions; the proportion of each T-RFs category (obligate anaerobes, facultative anaerobes, obligate aerobes) detected on media recommended for CF microbiology are highlighted in green. (TIF) [file pone.0022702.s003.tif]

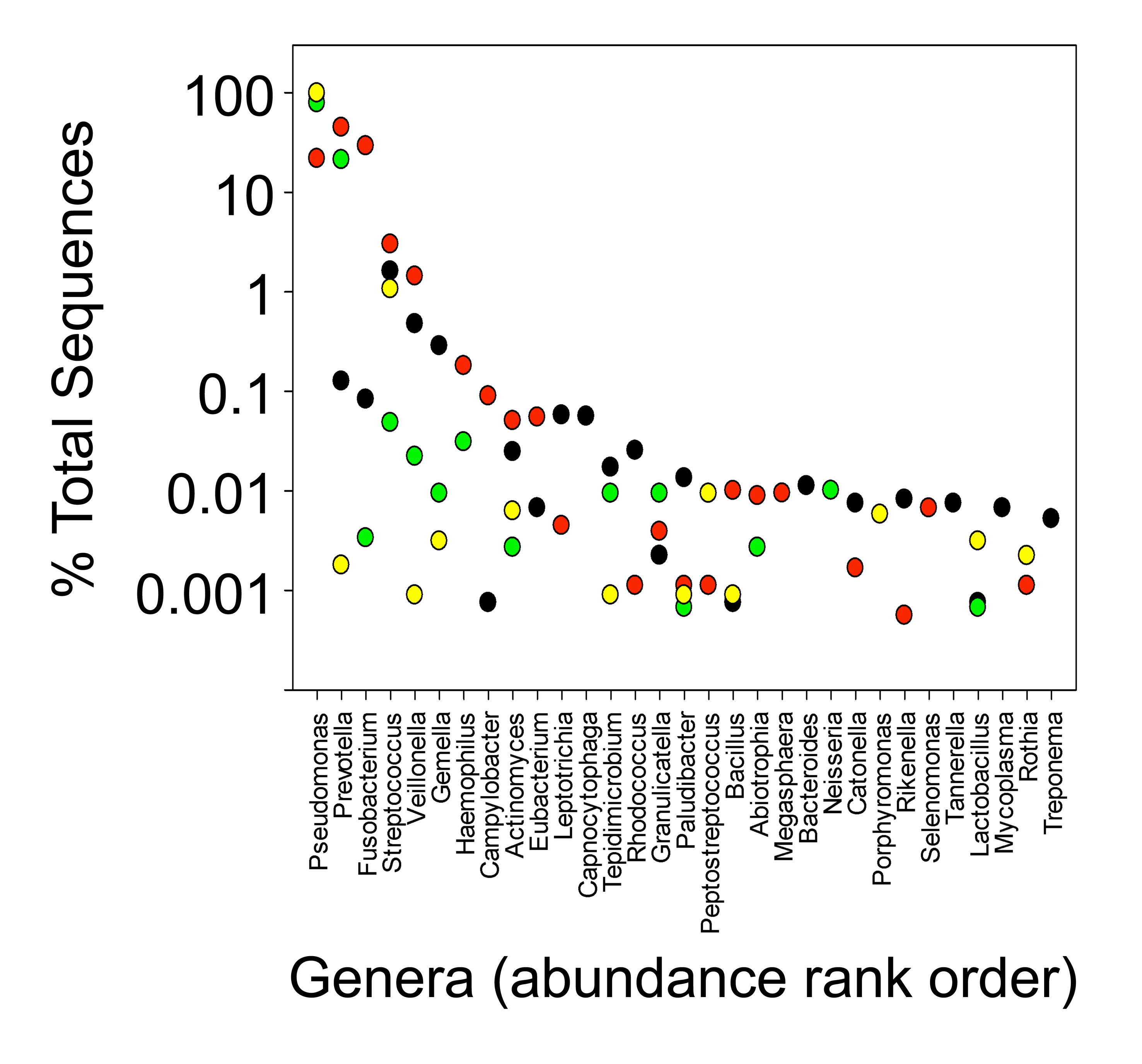

Supplement: Figure S4 — The most abundant genera detected from CF sputum by using deep 16S rRNA sequencing. The total number of sequences corresponding to each genus from all four patients was used to place genera in abundance rank order. Genera detected in greater than 0.001% of total sequences (greater than or equal to seven sequences in the total 689,422 generated from four patients) are shown. For each patient (red, green, yellow and black circles each illustrate an individual) the percent of the total patient-specific sequences represented by each genus is plotted. (TIF) [file pone.0022702.s004.tif]

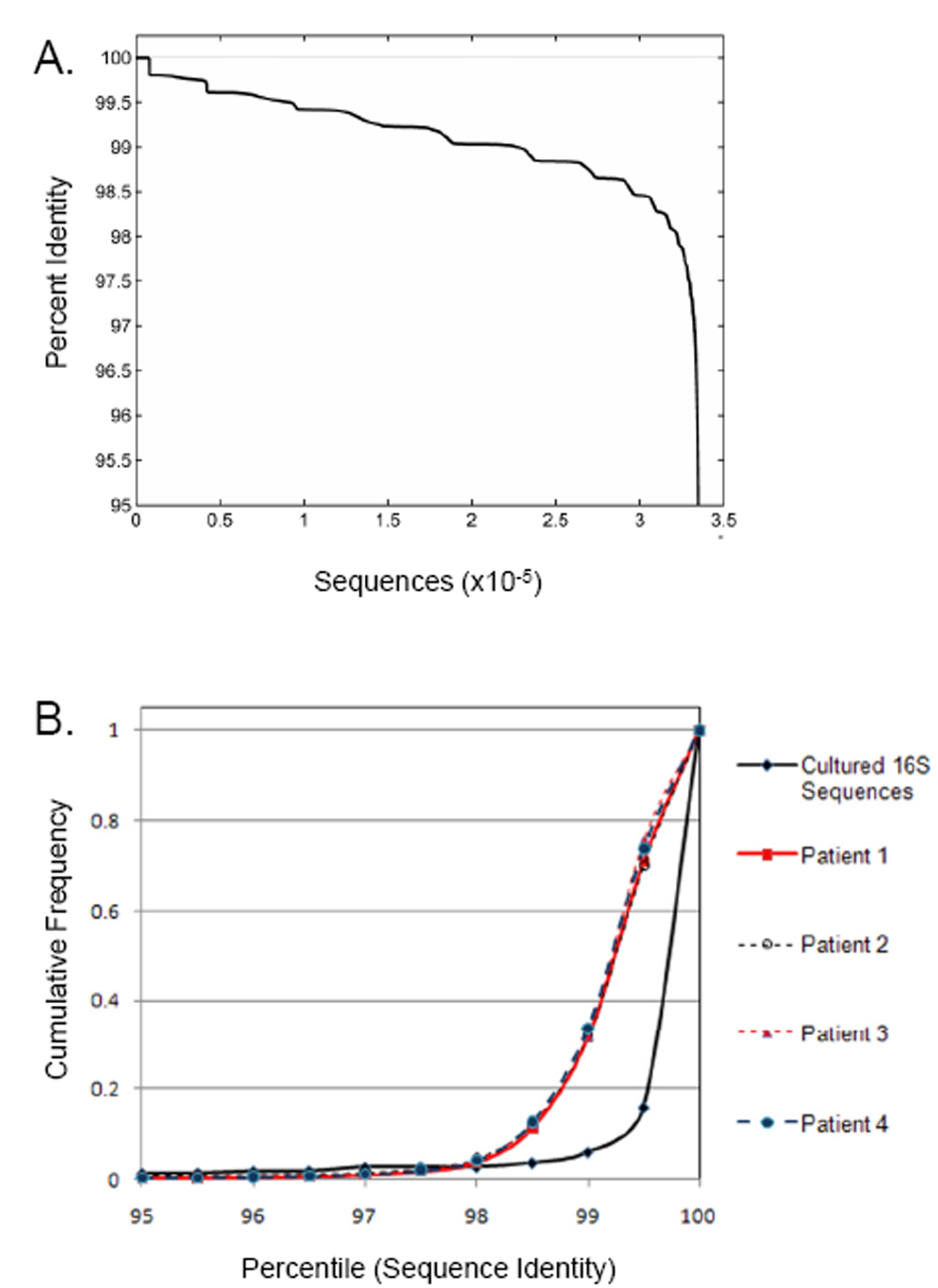

Supplement: Figure S5 — Pyrosequencing error results in a broader than expected distribution in 16S rRNA gene sequences and greater apparent sequence diversity. A. Distribution of P. aeruginosa 16S rRNA gene sequences pooled from the 4 independent 454 pyrosquencing runs of sputum samples. Theoretically all reads should be close to 100% match, however, the deviation from this represents sequencing error. Note this is for samples that have been filtered for poor sequence quality and chimeras. For these samples the estimated error rate is 0.9%. B. Cumulative frequency histogram for the P. aeruginosa sequences from each of the 4 independent 454 pyrosquencing runs of sputum samples along with the all of the P. aeruginosa 16S rRNA genes sequences from isolated organisms obtained by Sanger sequencing (n = 214). Note all four different pyrosequencing samples have similar distributions and only about 27% of the sequences fall in the 99.5–100% percentile (of sequence identity) compared to 85% of the Sanger sequenced samples. If the sequence diversity from the pyrosequencing for P. aeruginosa represented the true sequence variability, the cumulative histogram profiles would be expected to be different in each patient sample reflecting individual variability. (TIF) [file pone.0022702.s005.tif]

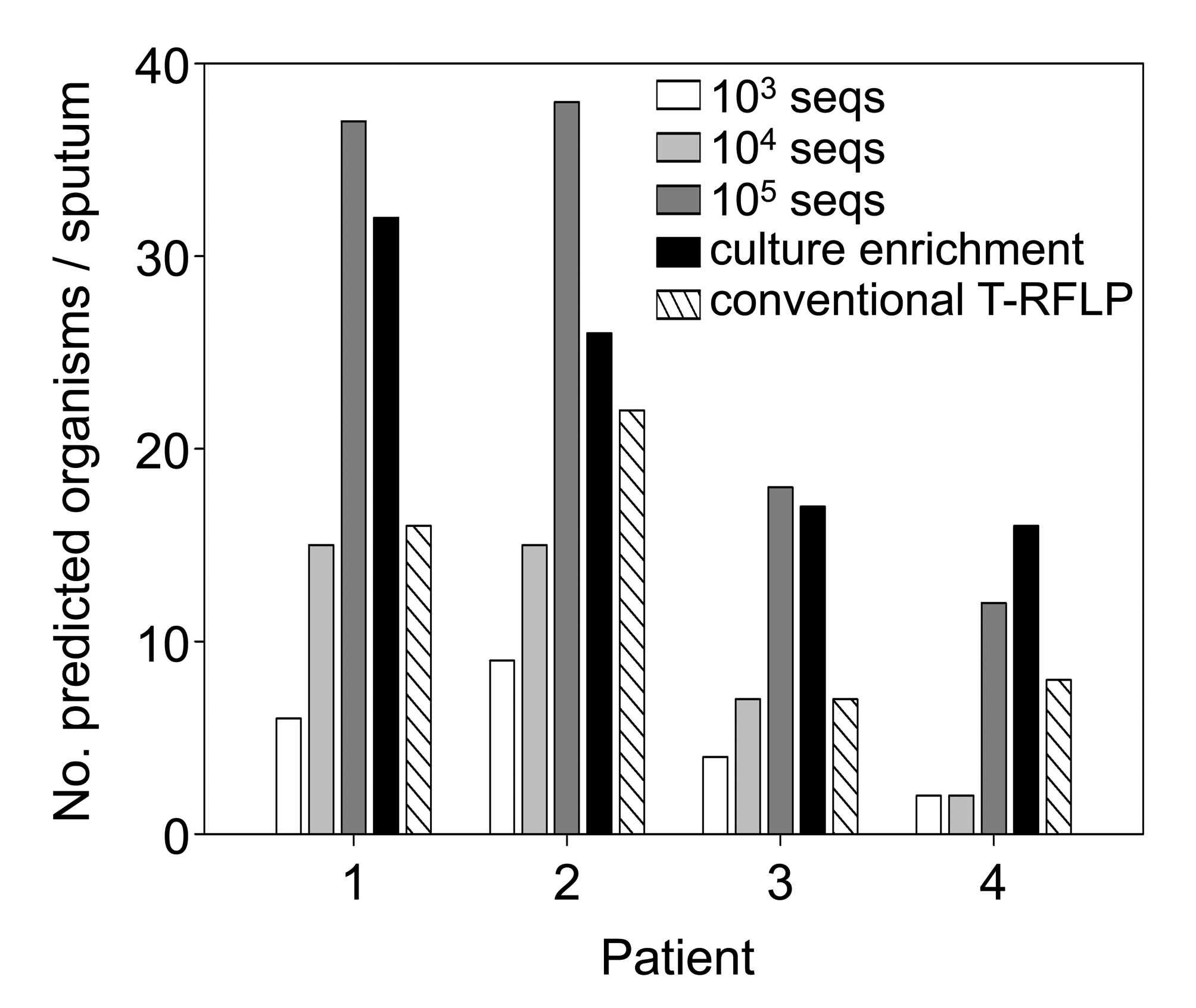

Supplement: Figure S6 — A comparison between the numbers of predicted organisms per sputum sample measured by conventional T-RFLP (determined by the number of T-RFs detected), culture-enrichment (as determined by 16S rRNA sequencing from enrichment pools) and by direct 16S rRNA sequencing from sputum at various depths (103 to 105 sequences per sputum). (TIF) [file pone.0022702.s006.tif]
