# Supplementary material for: Culture Enriched Molecular Profiling of the Cystic Fibrosis Airway Microbiome
Source: PLoS One. 2011 Jul 28;6(7):e22702. doi: 10.1371/journal.pone.0022702 (PMC3145661; doi:10.1371/journal.pone.0022702)
Supplement: Table S1 — Culture conditions used to generate the isolate collection. (DOC) [file pone.0022702.s007.doc]

**Table S1.** Culture conditions used to generate the isolate collection

| **Media** | **No. cultures** | **No. CO_2_ cultures** | **No. anaerobic cultures** | **Avg No. isolates* per culture** | **Max No. isolates**  **per culture** |
| --- | --- | --- | --- | --- | --- |
| BHI | 50 | 49 | 1 | 2.3 | 6 |
| CBA | 73 | 28 | 45 | 3 | 7 |
| CHOC | 16 | 0 | 16 | 2.4 | 4 |
| CNA | 63 | 19 | 44 | 2.5 | 8 |
| FAA | 51 | 0 | 51 | 3 | 6 |
| KVLB | 47 | 0 | 47 | 1.5 | 4 |
| MAC | 65 | 65 | 0 | 1 | 2 |
| McKAY | 281 | 281 | 0 | 2.2 | 6 |
| MSA | 38 | 32 | 6 | 1.1 | 2 |
| PEA | 42 | 0 | 42 | 2.83 | 6 |
| TSY | 25 | 25 | 0 | 2 | 6 |

* Isolates are defined as organisms recovered with distinct species-level classification based on 16S rRNA sequencing.
