# Supplementary material for: Culture Enriched Molecular Profiling of the Cystic Fibrosis Airway Microbiome
Source: PLoS One. 2011 Jul 28;6(7):e22702. doi: 10.1371/journal.pone.0022702 (PMC3145661; doi:10.1371/journal.pone.0022702)
Supplement: Table S2 — Characteristics of the culture collection generated by expanded culturing approaches (January 2006 to January 2010). (DOC) [file pone.0022702.s008.doc]

**Table S2.** Characteristics of the culture collection generated by expanded culturing approaches (January 2006 to January 2010)

| **Characteristic** | **Value** |
| --- | --- |
| No. patients represented | 117 |
| No. sputa planted | 351 |
| No. anaerobic cultures | 246 |
| No. 5% CO_2_ cultures | 505 |
| Mean no. of sputa per patient | 3 |
| Mean no. of species identified per sputum | 3.1 |
| Average number of species per patient | 6.9 |
| Max no. species recovered from a single sputum | 14 |
| Max no. species from a single patient | 33 |
