# Supplementary material for: Culture Enriched Molecular Profiling of the Cystic Fibrosis Airway Microbiome
Source: PLoS One. 2011 Jul 28;6(7):e22702. doi: 10.1371/journal.pone.0022702 (PMC3145661; doi:10.1371/journal.pone.0022702)
Supplement: Table S3 — Operational taxonomic units (3%) identified in the culture collection. (DOCX) [file pone.0022702.s009.docx]

**Table S3.** Operational taxonomic units (3%) identified in the culture collection

| **Family** | **Strain** | **16S rRNA Classification/**  **Genbank Accession No.** | **No. Isolates** |
| --- | --- | --- | --- |
| Actinomycetaceae | C909 | *Actinomces odontolyticus*/JF803601 | 45 |
|  | M166 | *Actinomyces naeslundii* II/JF803528 | 22 |
|  | C2398 | *Actinomyces naeslundii*/JF803596 | 8 |
|  | C1547 | *Actinomyces* sp./JF803583 | 6 |
|  | C601 | *Actinomyces viscosus/*JF803506 | 6 |
|  | C392 | *Actinomyces naeslundii II/*JF803503 | 5 |
|  | C549 | *Actinomyces graevenitizii/*JF803550 | 5 |
|  | C2358 | *Actinomyces radicidentis/*JF803591 | 3 |
|  | C84 | *Actinomyces naeslundii/*EF473995 | 3 |
|  | C1612 | *Actinomyces naeslundii/*JF803587 | 2 |
|  | C972 | *Actinomyces* sp./JF803567 | 2 |
|  | C59 | *Actinomyces* sp./EF474010 | 1 |
|  | C748 | *Actinomyces massiliensis/*JF803564 | 1 |
|  | C761 | *Actinomyces* sp./JF803565 | 1 |
| Aerococcaceae | C2444 | *Abiotrophia defectiva/*JF803600 | 4 |
| Bacillaceae | C1030 | *Bacillus sonorensis/*JF803556 | 5 |
|  | C1013 | *Bacillus subtilis/*JF803523 | 4 |
|  | C469 | *Bacillus anthracis/*JF803546 | 1 |
| Bacteriodaceae | C848 | *Bacteroides fragilis/*JF803518 | 2 |
| Bifidobacteriaceae | C834 | *Bifidobacterium longum/*JF803555 | 1 |
| Burkholderiaceae | C2298 | *Burkholderia cepacia/*JF803590 | 18 |
| Campylobacteraceae | C681 | *Campylobacter showae/*JF803571 | 1 |
| Carnobacteriaceae | C593 | *Granulicatella para-adiacens/*JF803551 | 34 |
|  | M141B | *Granulicatella adiacens/*JF803525 | 9 |
| Coriobacteriaceae | C2392 | *Atopobium parvulum/*JF803594 | 13 |
| Corynebacteriaceae | C2436 | *Corynebacterium durum/*JF803599 | 2 |
| Dermabacteraceae | C891 | *Dermabacter hominis/*JF803552 | 1 |
| Enterobacteriaceae | C517 | *Proteus mirabilis/*JF803548 | 3 |
|  | C783 | *Klebsiella oxytoca/*JF803553 | 24 |
| Enterococcaceae | M159 | *Enterococcus casseliflava/*JF803544 | 4 |
|  | M301 | *Enterococcus durans/*JF803535 | 1 |
|  | M352 | *Enterococcus faecalis/*JF803541 | 1 |
| Erysipelotrichaceae | C1107 | *Solobacterium moorei/* JF803577 | 1 |
| Flavobacteriaceae | C1549 | *Capnocytophaga gingivalis/*JF803584 | 1 |
| Fusobacteriaceae | C1135 | *Fusobacterium nucleatum/*JF803558 | 7 |
| Incertae Sedis XI | C456 | *Parvimonas micra/*JF803508 | 4 |
|  | C753 | *Finegoldia magna/*JF803573 | 1 |
| Lachnospiraceae | C1000 | *Oribacterium sinus/*JF803522 | 2 |
|  | C822 | *Eubacterium* sp./JF803516 | 2 |
| Lactobacillaceae | C797A | *Lactobacillus rhamnosus/*JF803554 | 46 |
|  | C2274 | *Lactobacillus gasseri/*JF803589 | 5 |
|  | C1170B | *Lactobacillus salivarius/*JF803559 | 4 |
|  | M474 | *Lactobacillus acidophilus/*JF803542 | 2 |
|  | C1241 | *Lactobacillus fermentum/*JF803560 | 1 |
| Micrococcaceae | C90_1 | *Rothia mucilaginosa/*JF803498 | 28 |
|  | C1865 | *Rothia denticariosa/*JF803588 | 14 |
|  | C1554 | *Kocuria polaris/*JF803585 | 3 |
|  | M63 | *Micrococcus luteus/*JF803533 | 1 |
| Moraxellaceae | C2393 | *Acinetobacter baumannii/*JF803595 | 1 |
| Neisseriaceae | C112 | *Neisseria flavescens/*JF803496 | 8 |
|  | C119 | *Neisseria elongata/*EF473988 | 2 |
|  | C1646 | *Neisseria sicca/*JF803581 | 1 |
| Pasteurellaceae | C2360 | *Haemophilus influenzae/*JF803592 | 4 |
| Peptostreptococcaceae | C1311 | *Peptostreptococcus stomatis/* JF803562 | 4 |
| Porphyromonadaceae | C1075 | *Porphyromonas* sp./JF803575 | 1 |
|  | C941 | unclassified Porphyromonadaceae/JF803519 | 1 |
| Prevotellaceae | C538 | *Prevotella melaninogenica/*JF803569 | 24 |
|  | C1065 | *Prevotella oris/*JF803574 | 20 |
|  | C1115 | *Prevotella histicola/*JF803578 | 19 |
|  | C1134 | *Prevotella denticola/*JF803557 | 13 |
|  | C579 | *Prevotella* sp./JF803513 | 8 |
|  | C823 | *Prevotella tannerae/*JF803517 | 2 |
|  | C960 | *Prevotella bivia/*JF803520 | 2 |
|  | C1116 | *Prevotella nanceiensis/*JF803579 | 1 |
|  | C1120 | *Prevotella salivae/*JF803580 | 1 |
|  | C503 | *Prevotella pallens/*JF803509 | 1 |
|  | C561 | *Prevotella melaninogenica/*JF803512 | 1 |
|  | C682 | *Prevotella intermedia/*JF803514 | 1 |
|  | C990 | *Prevotella* sp./JF803521 | 1 |
| Propionibacteriaceae | C649 | *Propionibacterium acnes/*JF803570 | 22 |
|  | C1250 | *Propionibacterium acidifaciens/*JF803561 | 3 |
| Pseudomonadaceae | C1602 | *Pseudomonas aeruginosa/*JF803586 | 211 |
|  | C402 | *Pseudomonas brenneri/*JF803504 | 1 |
| Staphylococcaceae | C365 | *Staphylococcus aureus/*JF803502 | 160 |
|  | M347 | *Gemella haemolysans/*JF803540 | 89 |
|  | M285 | *Gemella sanguinis/*JF803538 | 18 |
|  | C490 | *Staphylococcus epidermidis/*JF803547 | 9 |
|  | C350 | *Staphylococcus pasteuri/*JF803500 | 3 |
|  | C532 | *Staphylococcus hominis subsp hominis/*JF803549 | 3 |
| Rhodospirillaceae | M53 | *Inquilinus limosus/*JF803524 | 1 |
| Streptococcaceae | C2420 | *Streptococcus salivarius/*JF803598 | 247 |
|  | C419 | *Streptococcus constellatus /intermedius/*JF803505 | 148 |
|  | C91_2 | *Streptococcus mitis/*JF803497 | 133 |
|  | M143 | *Streptococcus parasanguinis II/*JF803526 | 100 |
|  | M247 | *Streptococcus infantis/*JF803530 | 82 |
|  | C1123 | *Streptococcus peroris/*JF803568 | 68 |
|  | C214 | *Streptococcus sanguinis/*JF803499 | 64 |
|  | M244 | *Streptococcus anginosus/*JF803543 | 62 |
|  | M147 | *Streptococcus cristatus/*JF803527 | 46 |
|  | M255 | *Streptococcus mutans/*JF803537 | 10 |
|  | C810 | *Streptococcus sp./*JF803566 | 9 |
|  | M338 | *Streptococcus dysgalactiae subsp equisimilis/*JF803539 | 4 |
|  | C42 | *Streptococcus oralis/*EF473999 | 3 |
|  | M319 | *Streptococcus agalactiae/*JF803536 | 3 |
|  | M492 | *Streptococcus pyogenes/*JF803531 | 3 |
|  | M627 | *Streptococcus sobrinus/*JF803545 | 3 |
|  | C629 | *Streptococcus infantis/*JF803507 | 2 |
|  | C1448 | *Streptococcus sp./*JF803582 | 1 |
|  | C362 | *Streptococcus sanguinis/*JF803501 | 1 |
|  | C701 | *Streptococcus* sp./JF803563 | 1 |
|  | M212 | *Streptococcus massiliensis/*JF803529 | 1 |
|  | M299 | *Streptococcus salivarius/*JF803534 | 1 |
|  | M595 | *Streptococcus parasanguinis/*JF803532 | 1 |
| Veillonellaceae | C2388 | *Veillonella parvula/*JF803593 | 12 |
|  | C2405 | *Veillonella atypical/*JF803597 | 7 |
|  | C1082 | *Megasphaera micronuciformis/*JF803576 | 3 |
|  | C535 | *Veillonella* sp./JF803510 | 1 |
|  | C546 | *Veillonella* sp./JF803511 | 1 |
|  | C710 | *Megasphaera* sp./JF803572 | 1 |
|  | C713 | *Dialister invisus/*JF803515 | 1 |
