# Supplementary material for: Culture Enriched Molecular Profiling of the Cystic Fibrosis Airway Microbiome
Source: PLoS One. 2011 Jul 28;6(7):e22702. doi: 10.1371/journal.pone.0022702 (PMC3145661; doi:10.1371/journal.pone.0022702)
Supplement: Table S4 — Taxonomic assignment results. Relative percentage of known (truth) dataset using 3 common taxonomic identification methods. (DOCX) [file pone.0022702.s010.docx]

**Table S4**. Taxonomic assignment results. Relative percentage of known (truth) dataset using 3 common taxonomic identification methods.

| **GENERA** | **Truth (%)** | **BLASTn** | **Qiime tax** | **RDP classification** |
| --- | --- | --- | --- | --- |
| *Phytoplasma* | *10.1836078* | *10.20679188* | *11.46876999* | *0.0* |
| *Streptomyces* | *7.571455612* | *7.398975526* | *8.356427201* | *8.691854117* |
| *Ktedonobacter* | *3.331440469* | *2.902675014* | *0.0* | *3.799956322* |
| *Campylobacter* | *2.93393905* | *2.826788086* | *3.325516947* | *3.406857392* |
| *Mycobacterium* | *2.820367216* | *2.693985961* | *3.176295033* | *3.253985586* |
| *Salmonella* | *2.233579406* | *2.23866439* | *3.282882115* | *2.314915921* |
| *Magnetobacterium* | *2.063221654* | *2.067918801* | *0.0* | *0.0* |
| *Levilinea* | *1.930721181* | *1.935116676* | *1.364314645* | *1.485040402* |
| *Burkholderia* | *1.76036343* | *1.764371087* | *1.982519719* | *2.009172308* |
| *Byssovorax* | *1.533219762* | *1.536710302* | *0.0* | *0.0* |
| *Bilophila* | *1.476433844* | *1.479795105* | *0.0* | *0.0* |
| *Bifidobacterium* | *1.419647927* | *1.460823373* | *1.598806225* | *1.637912208* |
| *WS3 (genus)* | *1.381790649* | *1.403908177* | *1.492219143* | *1.528718061* |
| *Corynebacterium* | *1.381790649* | *1.384936445* | *1.598806225* | *1.637912208* |
| *Achromobacter* | *1.381790649* | *1.384936445* | *1.662758474* | *1.703428696* |
| *Pasteurella* | *0.0* | *0.0* | *1.513536559* | *1.594234549* |
| *Helicobacter* | *0.0* | *0.0* | *1.342997229* | *1.375846255* |
| *Lactobacillus* | *0.0* | *0.0* | *0.0* | *1.419523914* |
