# Supplementary material for: Culture Enriched Molecular Profiling of the Cystic Fibrosis Airway Microbiome
Source: PLoS One. 2011 Jul 28;6(7):e22702. doi: 10.1371/journal.pone.0022702 (PMC3145661; doi:10.1371/journal.pone.0022702)
Supplement: Table S5 — Correlation R2 values of taxonomic assignment based upon a known dataset. (DOCX) [file pone.0022702.s011.docx]

**Table S5**. Correlation R2 values of taxonomic assignment based upon a known dataset.

| **Variables** | **Truth (%)** | **BLASTn** | **Qiime tax** | **RDP classification** |
| --- | --- | --- | --- | --- |
| *Truth (%)* | ***1*** | ***0.998*** | ***0.821*** | *0.130* |
| *BLASTn* | ***0.998*** | ***1*** | ***0.837*** | *0.114* |
| *Qiime tax* | ***0.821*** | ***0.837*** | ***1*** | *0.138* |
| *RDP classification* | *0.130* | *0.114* | *0.138* | ***1*** |
