# Supplementary material for: Culture Enriched Molecular Profiling of the Cystic Fibrosis Airway Microbiome
Source: PLoS One. 2011 Jul 28;6(7):e22702. doi: 10.1371/journal.pone.0022702 (PMC3145661; doi:10.1371/journal.pone.0022702)
Supplement: Table S6 — Correlation analysis P-value for taxonomic assignment based upon a known dataset. (DOCX) [file pone.0022702.s012.docx]

**Table S6.** Correlation analysis P-value for taxonomic assignment based upon a known dataset.

| **Variables** | **Truth (%)** | **BLASTn** | **Qiime tax** | **RDP classification** |
| --- | --- | --- | --- | --- |
| *Truth (%)* | ***0*** | ***< 0.0001*** | ***< 0.0001*** | *0.142* |
| *BLASTn* | ***< 0.0001*** | ***0*** | ***< 0.0001*** | *0.171* |
| *Qiime tax* | ***< 0.0001*** | ***< 0.0001*** | ***0*** | *0.129* |
| *RDP classification* | *0.142* | *0.171* | *0.129* | ***0*** |
