# Supplementary material for: Culture Enriched Molecular Profiling of the Cystic Fibrosis Airway Microbiome
Source: PLoS One. 2011 Jul 28;6(7):e22702. doi: 10.1371/journal.pone.0022702 (PMC3145661; doi:10.1371/journal.pone.0022702)
Supplement: Table S7 — Bacterial families detected in this study. (DOC) [file pone.0022702.s013.doc]

**Table S7.** Bacterial families detected in this study

| **Category** | **Family Name** |
| --- | --- |
| ­ALL | Actinomycetaceae |
|  | Aerococcaceae |
|  | Bacillaceae |
|  | Bacteroidaceae |
|  | Campylobacteraceae |
|  | Carnobacteriaceae |
|  | Clostridiales Family XI. Incertae Sedis |
|  | Coriobacteriaceae |
|  | Enterobacteriaceae |
|  | Flavobacteriaceae |
|  | Fusobacteriaceae |
|  | Lachnospiraceae |
|  | Lactobacillaceae |
|  | Micrococcaceae |
|  | Pasteurellaceae |
|  | Peptostreptococcaceae |
|  | Prevotellaceae |
|  | Pseudomonadaceae |
|  | Staphylococcaceae |
|  | Streptococcaceae |
|  | Veillonellaceae |
| Culture-enrichment only | Bacteriovoracaceae |
|  | Ruminococcaceae |
| Culture collection only | Bifidobacteriaceae |
|  | Corynebacteriaceae |
|  | Dermabacteraceae |
|  | Erysipelotrichaceae |
|  | Moraxellaceae |
|  | Propionibacteriaceae |
|  | Xanthomonadaceae |
| Deep Sequencing only | Comamonadaceae |
|  | Mycoplasmataceae |
|  | Rikenellaceae |
|  | Spirochaetaceae |
|  | Thermotogaceae |
| Culture-enrichment & Deep Sequencing | Bacillales Incertae Sedis |
|  | Clostridiaceae |
|  | Clostridiales Family XIII. Incertae Sedis |
|  | Eubacteriaceae |
|  | Mycobacteriaceae^1^ |
|  | Nocardiaceae |
|  | Planococcaceae |
|  | Rhizobiaceae |
| Culture-enrichment & Culture Collection | Burkholderiaceae |
|  | Enterococcaceae |
| Culture Collection & Deep Sequencing | Neisseriaceae |
|  | Porphyromonadaceae |
|  | Rhodospirillaceae |

_______________________________________________________________________________________________________

^1^ Cultured from CF sputum by clinical laboratory (Figure 1)
